# NGS-based *Aspergillus* detection in plasma and lung lavage of children with invasive pulmonary aspergillosis

**DOI:** 10.1038/s41525-025-00482-8

**Published:** 2025-03-17

**Authors:** Emmy Wesdorp, Laura Rotte, Li-Ting Chen, Myrthe Jager, Nicolle Besselink, Carlo Vermeulen, Ferry Hagen, Tjomme van der Bruggen, Caroline Lindemans, Tom Wolfs, Louis Bont, Jeroen de Ridder

**Affiliations:** 1 Center for Molecular Medicine, University Medical Center Utrecht, Utrecht, The Netherlands; 2 Oncode Institute, Utrecht, The Netherlands; 3 Hematopoietic stem cell transplantation, Princess Máxima Center for Pediatric Oncology, Utrecht, The Netherlands; 4 Westerdijk Fungal Biodiversity Institute, Utrecht, The Netherlands; 5 Department of Medical Microbiology, University Medical Center Utrecht, Utrecht, The Netherlands; 6 Institute for Biodiversity and Ecosystem Dynamics, University of Amsterdam, Utrecht, The Netherlands; 7 Department of Medical Microbiology, University Medical Centre Utrecht, Utrecht, The Netherlands; 8 Department of Pediatric Infectious Diseases and Immunology, Wilhelmina Children’s hospital, UMC Utrecht, Utrecht, The Netherlands

**Keywords:** High-throughput screening, Fungal infection

## Abstract

In immunocompromised pediatric patients, diagnosing invasive pulmonary aspergillosis (IPA) poses a significant challenge. Next-Generation Sequencing (NGS) shows promise for detecting fungal DNA but lacks standardization. This study aims to advance towards clinical evaluation of liquid biopsy NGS for *Aspergillus* detection, through an evaluation of wet-lab procedures and computational analysis. Our findings support using both CHM13v2.0 and GRCh38.p14 in host-read mapping to reduce fungal false-positives. We demonstrate the sensitivity of our custom kraken2 database, cRE.21, in detecting *Aspergillus* species. Additionally, cell-free DNA sequencing shows superior performance to whole-cell DNA sequencing by recovering higher fractions of fungal DNA in lung fluid (bronchoalveolar lavage [BAL] fluid) and plasma samples from pediatric patients with probable IPA. In a proof-of-principle, *A. fumigatus* was identified in 5 out of 7 BAL fluid samples and 3 out of 5 plasma samples. This optimized workflow can advance fungal-NGS research and represents a step towards enhancing diagnostic certainty by enabling more sensitive and accurate species-level diagnosis of IPA in immunocompromised patients.

## Introduction

Invasive mold disease (IMD) is a threat to immunocompromised children, especially those with hematological malignancies or undergoing hematopoietic stem cell transplantation (HSCT)^1^. Despite receiving antifungal prophylaxis, breakthrough IMD can still occur, with an incidence of up to 20%^2–6^, with *Aspergillus* being the most common cause of IMD^7^. Early and accurate identification of fungal pathogens is crucial for tailoring antifungal treatment, especially as diverse fungal pathogenic species require different antifungal treatments. For aspergillosis, an azole is recommended as first-line treatment, whereas for mucormycosis amphotericin B is the first-choice treatment^8^.

The current diagnostic toolbox for IMD includes radiologic imaging, microbiological bronchoalveolar lavage (BAL) fluid analysis (i.e., culture, antigen testing and PCR) and antigen testing on serum. While valuable^9^, these microbiological tests have limited sensitivity, particularly as prior antifungal treatment — common in pediatric patients — compromises their performance. For instance, BAL PCR shows a sensitivity of 0.17% (95% CI, 0.05–0.45), while BAL galactomannan has a sensitivity of 60%^10,11^. Additionally, the galactomannan antigen test lacks species level identification, which is crucial in the differentiation between invasive aspergillosis from invasive non-*Aspergillus* species. Therefore, there is an urgent clinical need to expand the diagnostic toolbox for early, sensitive and accurate species level diagnosis, preventing disease progression, improving patient outcomes and avoiding unnecessary exposure to prolonged toxic antifungal therapy.

Microbial next-generation sequencing (NGS) detects microbial DNA of pathogens in patients with infectious diseases and holds promise for IMD diagnosis^12–16^. Microbial NGS enables species-level identification of pathogens and is regarded as sensitive when applied to BAL samples^16^, while its sensitivity in plasma appears to depend on the specific pathogen responsible for the IMD^15,16^. Yet, the preferred microbial NGS workflow for pediatric IMD diagnosis is unclear and multiple technical gaps exist. Computationally, there is a lack of standardization on taxonomy classification for fungal identification in samples from patients with IMD. Specifically, the impacts of reference database composition, genomic sequence processing (i.e., masking low-complexity and contaminated regions) and threshold settings for sequencing read classification in fungal diagnosis remain unclear. Given that only a small fraction of short-read sequences corresponds to potential pathogens, any taxonomic misassignment due to suboptimal computational methods or parameters can greatly affect pathogen identification and subsequent therapeutic decisions. Such misclassification may occur when reads coincidentally match to multiple genomes in the reference database or persistently match to an incorrect genome or no genome at all leading to false positive or false negative outcomes^17^.

Additionally, it remains unclear what the optimal wet-lab strategy is to maximize fungal DNA yield. Specifically, little is known about the impact of sample source, DNA type, and DNA isolation method on the success of fungal diagnosis. Although traditionally whole-cell DNA (wcDNA) sequencing has been applied to samples collected near the infection sources, like BAL fluid, recent research indicates that BAL fluid cell-free DNA (cfDNA) sequencing may outperform wcDNA sequencing in pulmonary infections^18^. At the same time, plasma microbial cfDNA sequencing has also shown potential in mostly small cohort studies involving adult IMD patients^12,13,15,16^, and one pediatric study^14^, highlighting its potential but also emphasizing the need for further investigation. Methods like DNA isolation and adapter ligation can also affect fungal DNA abundance. Although single-stranded (ss) sequencing libraries are preferred for e.g., bacterial and viral cfDNA over double-stranded (ds) ligation-based DNA library preparation^19^, further exploration through comparative evaluation has not yet been done to determine the optimal approach for recovering fungal mold DNA from liquid biopsies.

**Fig. 1:**
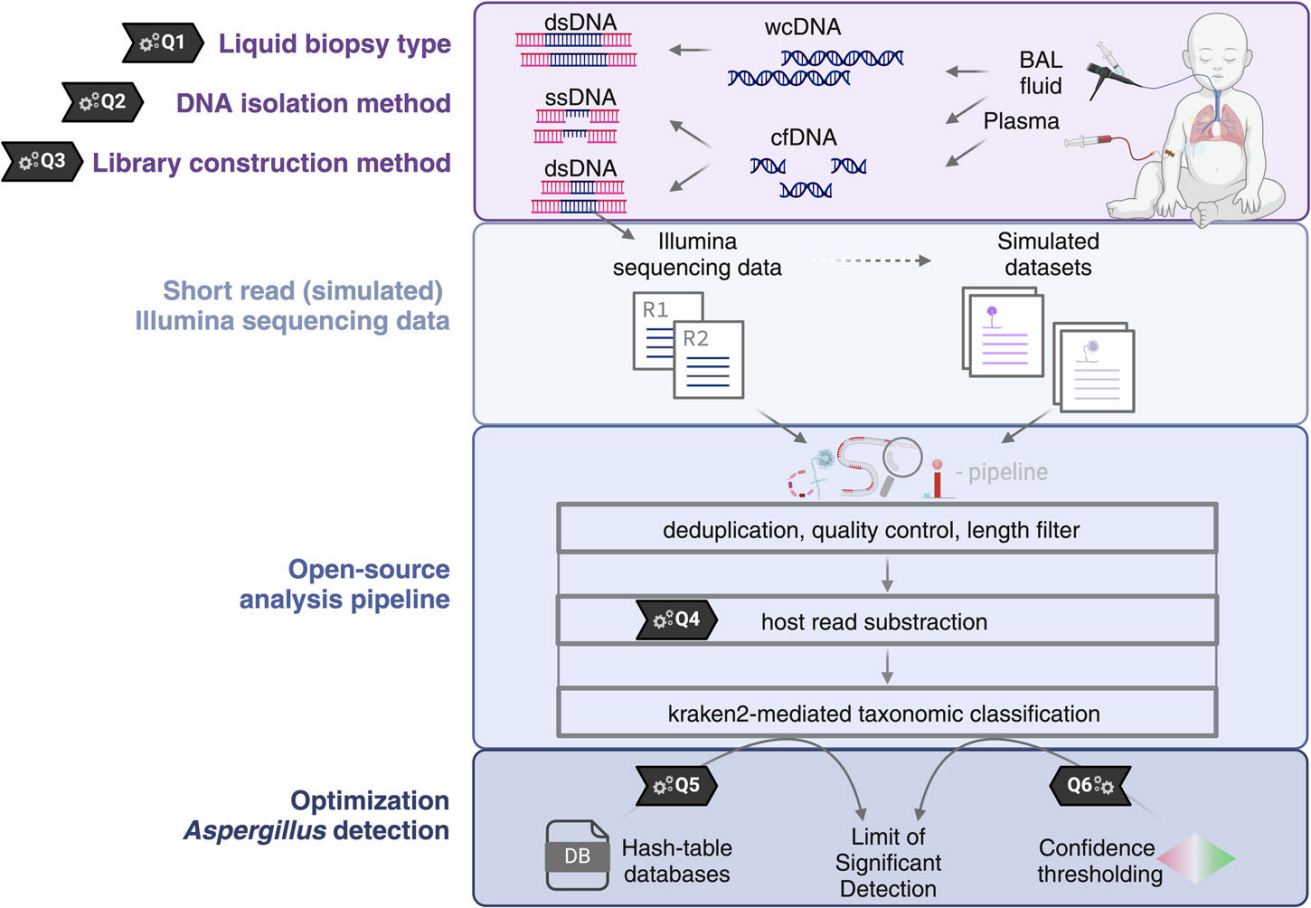
NGS library strategies and the cfSPI open-source workflow for *Aspergillus* detection. Our comparative study employs Illumina shotgun sequencing to enhance *Aspergillus* DNA detection in liquid biopsies from pediatric immunocompromised patients. We set to optimize six key steps (Q#1-6) for microbial NGS-based *Aspergillus* diagnostics in IMD patients. We compared two cell-free DNA NGS library preparation strategies — single-stranded (ss) and double-stranded (ds) ligation — across plasma and bronchoalveolar lavage (BAL) fluid samples (Q#1), alongside a comparison of BAL cfDNA to whole-cell DNA (wcDNA) NGS (Q#2-3). After sequencing, we employ the open-source cell-free DNA pathogen identification workflow known as the **c**ell-**f**ree DNA **S**ingle-strand **P**athogen **I**dentification pipeline (**cfSPI**), which is tailored for detecting *Aspergillus* species. Within the cfSPI pipeline, we conduct quality control of sequencing data followed by host-read subtraction (Q#4) and taxonomic classification using *kraken2* with various hash-table genome reference databases (Q#5) and confidence thresholds (Q#6). To assess the accuracy of (*Aspergillus*) read classification, we further simulate short-read Illumina sequencing data. In our Limit of Significant Detection analysis, we investigate how hash-table database complexity and confidence thresholds impact the theoretical minimum number of molecules per million needed to detect significantly elevated *Aspergillus* taxon above the background levels in control patient samples. All six key steps underwent optimization through comparative experimental testing. Illustrations was created using BioRender (https://BioRender.com/o72k148).

In this study, we focus on the detection of *Aspergillus*, the predominant pathogen associated with pulmonary IMD. We aim to close above-mentioned technical knowledge gaps by optimizing six key steps through comparative experimental testing (Q#1-6; detailed in Fig. 1). We introduce a refined wet-lab strategy together with an open-source cfDNA pathogen identification workflow, referred to as **c**ell-**f**ree DNA **S**ingle-strand **P**athogen **I**dentification pipeline (**cfSPI**). cfSPI is optimized for the detection of *Aspergillus* species with minimal false positives taxonomic mislabeling and maximum accuracy of true positive detections. The cfSPI pipeline uses paired-end Illumina sequencing data and incorporates host genome mapping. Unmapped reads are subsequently classified using *kraken2*^20^, with enhancements such as an improved reference database through *dustmasking* and *cleanup*^20,21^, and an optimal confidence threshold (CT) for classification accuracy. We show that these factors, all refined in this study, can impact *Aspergillus* classification accuracy^17,22–25^.

Finally, as a proof-of-principle, we applied cfDNA sequencing with cfSPI to seven pediatric patients (7 BAL fluid; 5 plasma) with invasive pulmonary aspergillosis (IPA) and eighteen external controls (9 BAL fluid; 9 plasma), successfully detecting *Aspergillus* species in the majority (6/7) of these IPA cases. This work establishes the groundwork for large cohort evaluations of the accuracy and sensitivity of cfDNA NGS for *Aspergillus* diagnosis in suspected patients, ultimately contributing to the potential implementation of NGS in the IMD diagnostic work-up.

## Results

cfSPI is an open-source pipeline optimized for accurate *Aspergillus* detection. The pipeline processes paired-end Illumina sequencing data through quality filtering, host genome mapping, and classification using *kraken2*, a high-performance DNA-to-DNA tool leveraging 31-mer matches against a reference database. Each step is carefully fine-tuned, as described in the next section. To validate the results produced by cfSPI, we generated 87 simulated Illumina sequencing cfDNA datasets (55 *Aspergillus*, 7 *Penicillium*, 25 other fungi). Moreover, we Illumina sequenced samples of seven probable invasive pulmonary aspergillosis patients and 18 external controls to further showcase the utility of cfSPI on patient data.

### Optimizing host read subtraction and kraken2 database composition for the cfSPI pipeline

**Fig. 2:**
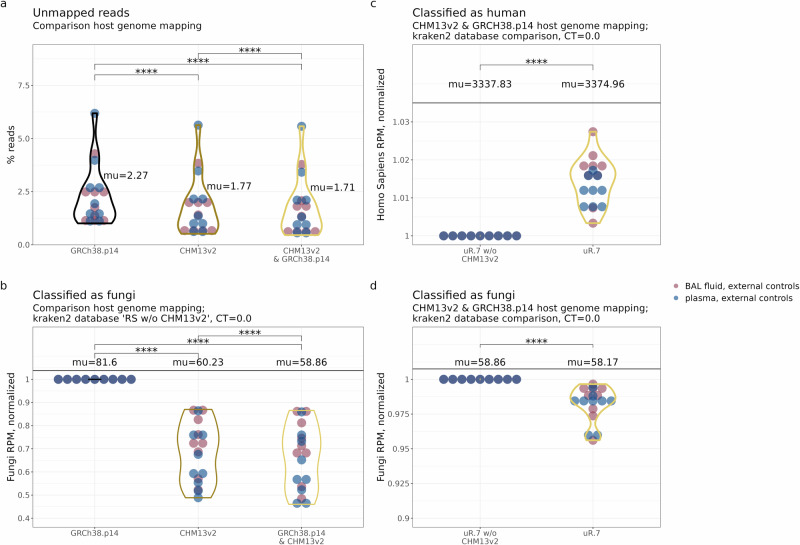
Determining the impact of CHM13v2 host genome mapping for optimizing microbial read quantification in control samples. **a** Percentage of reads remaining after mapping to the human reference genome (i.e., % unmapped reads) using the cfSPI workflow. Reads were mapped either to human reference genome GRCh38.p14, CHM13v2, or a combination of these two. **b** Fractional abundance of fungi (kingdom) *kraken2* classified reads (CT = 0, kraken2’s default), after subtracting host reads via reference genome mapping, normalized to the old version of the human genome assembly (GRCh38.p14). Fractional abundance of **c**. human (species) and **d** fungi (kingdom) classified reads when utilizing the ‘CHM13v2-containing uR.7’ or ‘uR.7’ database for kraken2 taxonomic classification (CT = 0, kraken2’s default) after dual-mapping to the host genome, normalized to ‘uR.7’. In **a**–**d**, each data point represents one control sample (9 BAL; 9 plasma), with colors indicating the sample type. Mean values are denoted as ‘mu’. Statistical analysis included one-tailed paired t-tests with Bonferroni correction (****, p ≤ 0.0001).

Detecting *Aspergillus*-derived DNA fragments from (cf)DNA sequencing data is a ‘needle-in-a-haystack’ challenge, where the vast majority of DNA reads will be derived from the host. For this reason, host-read subtraction through mapping to the host genome is a critical initial step in cfSPI to minimize the risk of overestimating microbial counts. Previous work already highlighted that improper subtraction can inflate bacterial counts^23^. There are a number of frequently used human genome versions, such as GRCh38.p14 and CHM13v2, which vary in completeness. The impact of mapping to these different genome versions on detecting fungal-derived reads in liquid biopsies remains unclear. To evaluate this, we mapped the sequencing reads from our external control samples (9 BAL; 9 plasma) to reference genomes GRCh38.p14 or CHM13v2. In addition, we performed *dual-mapping* meaning mapping was performed to a combined reference containing both GRCh38.p14 and CHM13v2. Our results revealed that both mapping to CHM13v2 and dual-mapping strategies significantly reduced the fraction of unmapped reads compared to mapping to GRCh38.p14, with rates dropping from 2.27% to 1.77% and 1.71%, respectively (Fig. 2a).

Unmapped reads were taxonomically classified using kraken2 (see *Methods*), using the default confidence threshold (CT = 0). We found that fungal-classified reads dropped from 81.60 reads per million (RPM) with GRCh38.p14 to 60.23 RPM and 58.86 RPM with CHM13v2 and dual-mapping (Fig. 2b)^24^. These findings indicate that including CHM13v2 is essential to prevent inflated fungal counts. Overall, dual-mapping emerges as the preferred strategy for reducing misclassifications to the fungal kingdom.

Next, we focused on optimizing the kraken2-mediated taxonomic classification of non-human reads. To address the possibility of human reads remaining unidentified during host mapping, we evaluated whether incorporating CHM13v2 into the standard NCBI RefSeq database used by *kraken2*, which traditionally included only GRCh38.p14, could help reduce misclassifications (see Supplementary Fig. 2 for database details). Indeed, inclusion of CHM13v2 (which we refer to as database ‘uR.7’) increased reads labeled as human (Fig. 2c, CT = 0, the default setting in kraken2; Supplementary Fig. 3a CT = 0.8), even after dual-mapping, while reducing fungal (Fig. 2d), but not other microbial counts (Supplementary Fig. 3b-c). Additionally, omission of CHM13v2 from the hash-table database led to misclassification of reads as microbial (Supplementary Fig. 4a), including as *Aspergillus* (Supplementary Fig. 4b). Therefore, in our cfSPI pipeline, we utilize *kraken2* databases that include both CHM13v2 and GRCh38.p14 to further mitigate the risk of misclassifying human-derived reads as fungal or microbial taxa.

**Fig. 3:**
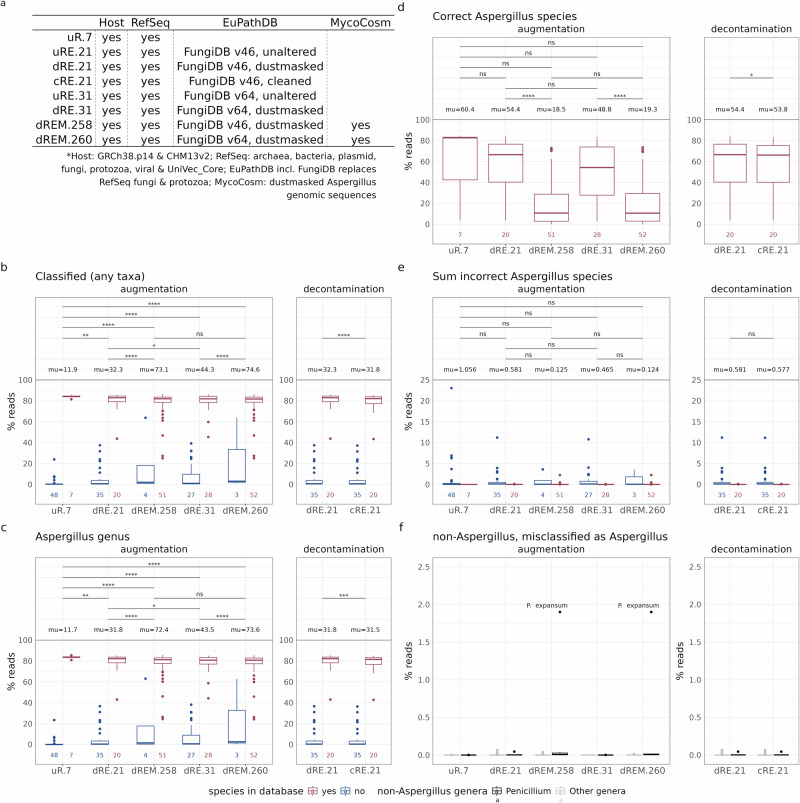
Database-dependent taxon detection in simulated *Aspergillus* samples. **a** *Kraken2* hash-table database composition overview (for comprehensive details on database composition, see Supplementary Fig. 2 and for details on database construction see *Methods* and Supplementary Data 5). Boxplots displaying the results of the classification of simulated *Aspergillus* samples, including **b**. the overall classification rate (e.g., percentage reads classified to any taxon) as well the percentage of reads classified at the **c**. *Aspergillus* genus level, **d**. to correct *Aspergillus* species, and **e**. cumulative percentage to incorrect *Aspergillus* species. Read classification percentages are shown across databases (x-axis). **f**. Displaying the results of the classification of *Penicillium* (n = 7) and other fungal genera (n = 25). The boxplots show the percentage of non-*Aspergillus* simulated reads that were erroneously classified as *Aspergillus*. **b-f**. The CT was set at 0.8 for *kraken2*-based classification, to increase precision^20^. In **b-e**. the effects of hash-table database augmentation (on the left) and decontamination (on the right) on accuracy are tested via a one-tailed t-test with Bonferroni correction (*, p ≤ 0.05; **, p ≤ 0.01; ***, p ≤ 0.001; ****, p ≤ 0.0001; ns, p > 0.05) and the mean percentages are denoted as ‘mu’. The boxplot bounds in **b-f**. represent the 25th and 75th percentiles, with the center line indicating the median. Whiskers extend to 1.5 times the interquartile range.

The default kraken2 NCBI RefSeq database (‘uR.7 w/o CHM13v2’) traditionally contains only seven out of the over 300 known *Aspergillus* species (Supplementary Fig. 2a-b), leading to inadequate *Aspergillus* classification. Specifically, 88.1% of reads from 55 simulated *Aspergillus* datasets remained unclassified (i.e., not classified to any taxa; Fig. 3b) when using a CT of 0.8, due to the absence of these species in the database (Fig. 3b). With the aim to enhance (species-level) classification of *Aspergillus*, we replaced the fungal genomes in the uR.7 database with **c**leaned, **d**ustmasked or **u**naltered fungal sequences from EuPathDB^26^ and MycoCosm^27^ (Fig. 3a; database details in Supplementary Fig. 2, *Methods*, and *Aspergillus* species in Supplementary Data 3). This resulted in new *kraken2* databases: **u**RE.**21**, **d**RE.**21**, which include **21** *Aspergillus* species), and **u**RE.**31**, **d**RE.**31**, which include **31** *Aspergillus* species. As well as: **d**REM.**258** and **d**REM.**260**, which include **258** and **260** *Aspergillus* species, respectively.

When evaluating the performance of these new *kraken2* databases, we first assessed the effects of dustmasking and cleanup, followed by the impact of database augmentation on the classification of simulated Illumina cfDNA *Aspergillus* datasets. Using a cleaned or dustmasked database proved crucial for preventing taxonomic misassignments of *Aspergillus*. We found a slight but significant reduction in overall classification rates (7.0–8.2%) and true positives at the genus (6.7–7.6%) and species levels (8.1–9.1%) with a CT of 0.8 (Supplementary Fig. 5a-c, e-g). However, classification accuracy improved significantly at CT values < 0.2 (Supplementary Fig. 5h), reaching a 0.30–0.38% reduction in false positives (Supplementary Fig. 5d). These findings suggest that masking unreliable sequences in reference genomes is highly recommended. Second, we evaluated the impact of database augmentation on classification accuracy across different taxonomic levels. By incorporating fungal sequences from EuPathDB^26^ and MycoCosm^27^ into the *kraken2* database, we identified a trade-off between database size and classification accuracy. Specifically, we observed the followings trends linked the extended databases: improved overall classification rates (Fig. 3b), increased true positives at the genus level (Fig. 3c), a broader range of detectable *Aspergillus* species (Supplementary Data 4), and a reduced false positive rate during species classification (Fig. 3e; Supplementary Fig. 6). However, we also observed a lower percentage of reads classified at the species level (Fig. 3d). This while, misclassification of *Penicillium* as *Aspergillus* generally remained low but increased when expanding the database with MycoCosm genomes, particularly in dREM.260, where we noted 1.97% misclassification at the genus level and 1.90% at the species level (Fig. 3f).

Overall, we conclude that medium-sized databases with 21 to 31 *Aspergillus* genomes are optimal for species level detection, while larger databases (258 to 260 genomes) excel in broader genus level detection. The curated cRE.21 database thereby demonstrated the highest sensitivity for species level detection (mean 53.8%; Fig. 3b), while dREM.260 excelled in genus level detection (mean 73.6%; Fig. 3c). Consequently, the cfSPI pipeline uses cRE.21 for species identification and dREM.260 for genus identification.

### Optimizing sample workup for fungal NGS

Sample preparation can impact the sensitivity of shotgun microbial NGS for *Aspergillus* detection. We isolated cfDNA and wcDNA from BAL fluid and cfDNA from plasma, constructing sequencing libraries using either single-stranded (ss) or double-stranded (ds) ligation methods (for schematic overview see Fig. 1; for details see *Methods*). Sequencing was performed on 60 libraries (see Supplementary Data 2). With the optimized computational workflow, elevated fungal counts are interpreted as indicative of improved fungal DNA retrieval rather than false positive observations.

**Fig. 4:**
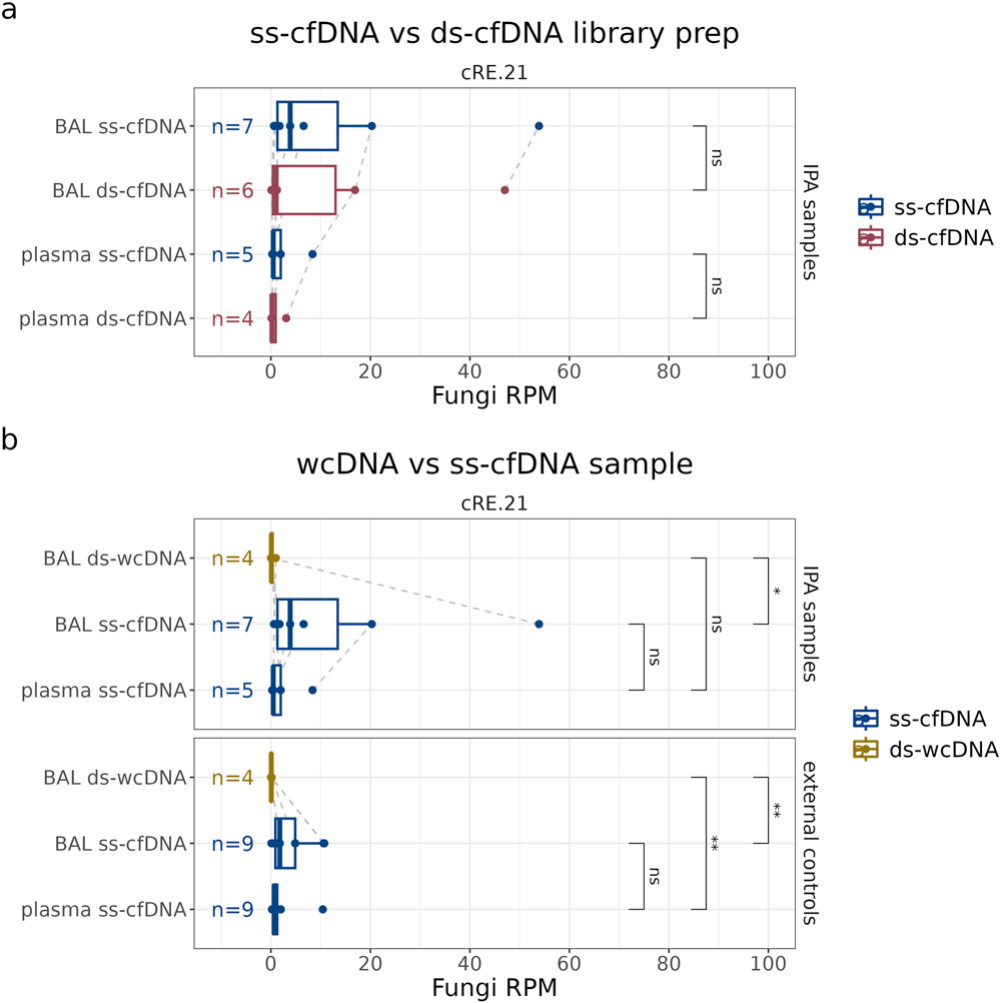
ss-ligation of cfDNA most effective in retrieving fungal DNA. **a, b** Boxplots showing the fungal (kingdom) fractional abundance in RPM, determined by cRE.21-mediated taxonomic classification (CT = 0.8). This analysis was conducted, where possible, for both IPA patients and external control samples. **a** Comparison fungal fractional abundance in sequencing libraries, emphasizing the impact of ss- versus ds-ligation based library preparation. **b** Comparison of BAL pellet wcDNA to supernatant cfDNA sequencing. **a, b** The boxplot bounds represent the 25th and 75th percentiles, with the center line indicating the median. Whiskers extend to 1.5 times the interquartile range. Statistical significance is evaluated through a one-tailed Wilcoxon rank test with Bonferroni correction (*, p ≤ 0.05; **, p ≤ 0.01; ns, p > 0.05). Dotted lines connect sequenced libraries derived from samples collected from the same patient.

Previous work, focusing on the cfDNA in plasma, demonstrated that ss-ligation produced a higher yield of short (<100 bp) microbial cfDNA compared to dsDNA ligation^19^. We assessed if the same holds true for fungal and *Aspergillus* cfDNA in our sample set of IPA patient- and external control samples, while making use of the cRE.21 database and a CT of 0.8. A noticeable trend in all samples suggests that ss-ligation generally resulted in elevated fungal (Fig. 4a) and *Aspergillus* (Supplementary Fig. 7a) relative abundance in both plasma and BAL samples (n.s., Wilcoxon rank-sum test; p > 0.05). We observed *Aspergillus* reads in almost all of these liquid biopsy samples (Supplementary Fig. 7c). In addition, ss-ligation resulted in a narrower library-size range compared to ds-ligation (Supplementary Fig. 8), circumventing DNA yield-reducing bead-based size selection (i.e., elimination of DNA molecules >700 bp; see *Methods* and Supplementary Data 2). Together, these results confirm that ss-cfDNA NGS is more effective than ds-DNA NGS for the recovery of fungal DNA from liquid biopsy samples^19^.

Further analysis of ss-cfDNA workup revealed a significantly higher fungal (Fig. 4b) and *Aspergillus* (Supplementary Fig. 7b) abundance in the cfDNA when compared to wcDNA BAL sequencing (p ≤ 0.05, one-tailed Wilcoxon’s rank with Bonferroni correction). This difference could not be attributed to a difference in sequencing-depth as there is no correlation between the total read count and the relative number of fungal reads in our external control samples (Supplementary Fig. 7e; p > 0.05, Pearson correlation). These observations thus confirm earlier reports^18,28^ that cfDNA contains a relatively higher fraction of fungal DNA molecules than wcDNA. Moreover, our study reveals that fungal counts in IPA patient BAL fluid ss-cfDNA samples are, on average, 5.4x higher than the relative fungal abundance in IPA patient plasma samples (n.s., one-tailed Wilcoxon’s rank with Bonferroni correction). This may be attributed to the direct sampling of BAL fluid at the presumed site of the *Aspergillus* infection. Taken together, the ss-cfDNA library preparation method results in higher fungal DNA relative abundance from both BAL and plasma samples, thereby exhibiting slightly higher abundances in BAL fluid.

### Optimizing confidence thresholding: analyzing theoretical minimum for *Aspergillus* detection

**Fig. 5:**
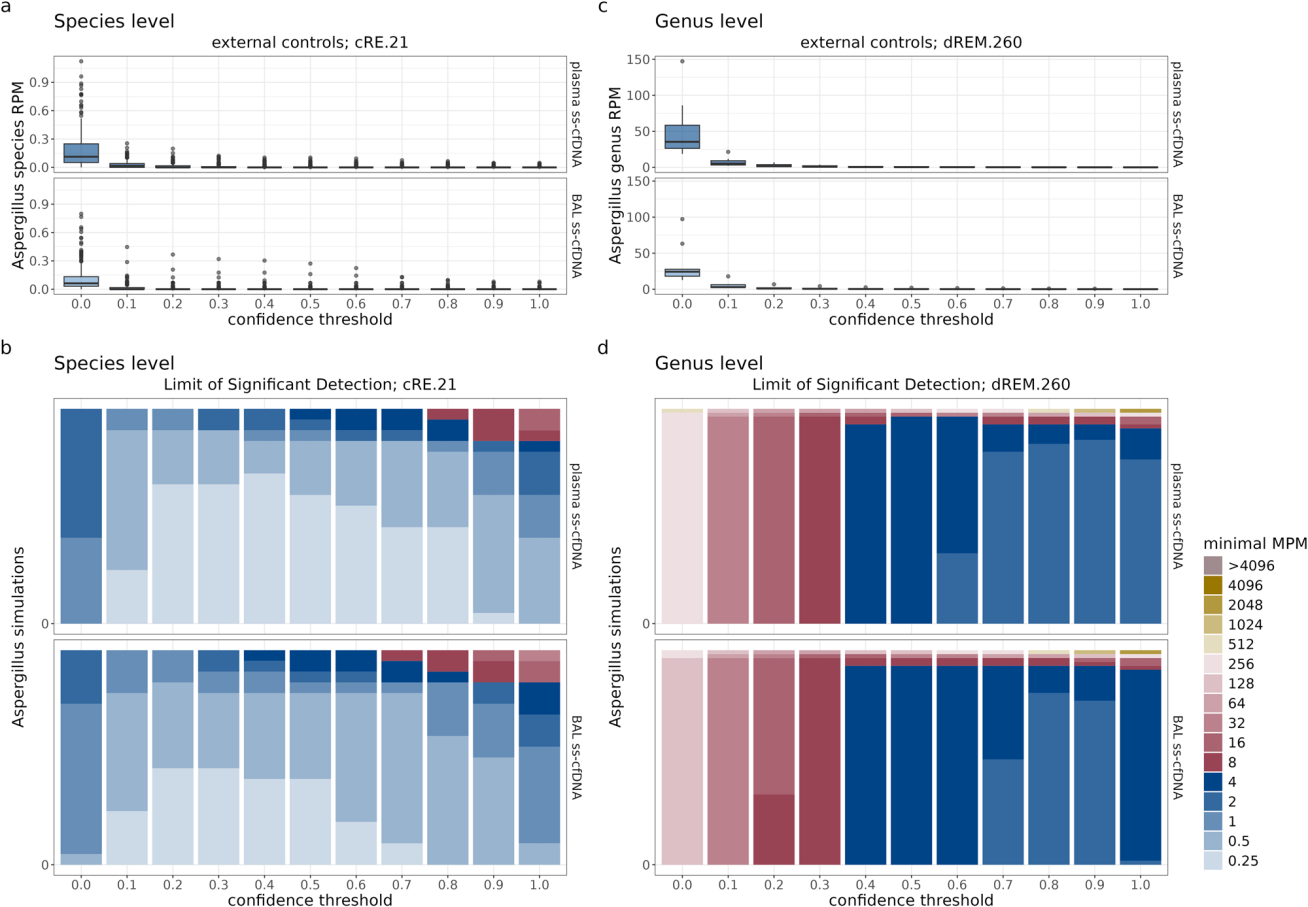
Computational analysis theoretical minimum fraction required for *Aspergillus* detection to optimize database and parameter selection. The fractional **a**. species level (cRE.21-mediated classification) and **c**. genus level (dREM.260-mediated classification) abundance of *Aspergillus* (in RPM) delineated for plasma (n = 9) and for BAL fluid (n = 11) external control samples. In **a**,**c**. the boxplot bounds represent the 25th and 75th percentiles, with the center line indicating the median. Whiskers extend to 1.5 times the interquartile range. Leveraging the background levels from our external control samples and classification rates derived from simulations (not shown), we calculated the theoretical minimum fraction of *Aspergillus* molecules (in molecules per million; MPM) necessary for the detection of significantly elevated *Aspergillus* levels above the control background, as visualized in **b**,**d**. The minimum of *Aspergillus* MPM was computed both at the **b**. species level (cRE.21-mediated) and **d**. genus level (dREM.260-mediated), at a theoretical sequencing depth of 70 million (M) reads/sample (for details, see *Methods*). The subsequent confidence threshold (CT) parameter optimization was based on the highest fraction of observation at <4 MPM.

After application of the cfSPI pipeline some *Aspergillus* counts were above zero in control samples (see Supplementary Fig. 7b,d for *Aspergillus* prevalence and Supplementary Fig. 7b for relative abundance in external control samples). These *Aspergillus* background levels must thus be considered when conducting diagnostic testing. Overall, background levels in control samples were higher at the genus (dREM.260-mediated) than at the species level (cRE.21-mediated), and higher in plasma samples compared to BAL samples (mean 1.5x and 1.2x using the cRE.21 and dREM.260, respectively) (Fig. 5a,c). Furthermore, these background levels were influenced by classification confidence thresholding (Fig. 5a,c). Acknowledging that both computational choices thus directly affect our ability to detect elevated *Aspergillus* DNA levels in patients suspected of a pulmonary fungal infection, we developed a methodology to explore the relationship between fungal background levels, classification rates in simulated datasets, and theoretical Limits of Significant Detection (LoSD).

In this LoSD analysis, we computed the theoretical minimum number of molecules per million (MPM) necessary for the detection of significantly elevated *Aspergillus* taxa above the control background, i.e., the background levels observed in immunocompromised pediatric patients without suspicion of a fungal infection (see *Methods* for details). Based on the previously observed species level classification rates (Fig. 3), our hypothesis was that the cRE.21 database would outperform the dREM.260 for detecting *Aspergillus* in clinical samples. And indeed, our LoSD analysis showed that species detection with the dREM.260 necessitated a substantially higher cfDNA load than species level detection with the cRE.21 (Fig. 5b; for details see Supplementary Fig. 9a).

Recognizing the interplay between database and CT choices in our LoSD analysis, we established the most optimal CT for each database. Species level cRE.21-mediated detection was most sensitive when employing a *kraken2* CT of 0.4, while genus level dREM.260-mediated detection required a CT of 0.9 (Fig. 5b,d). These findings are based on plasma, where fungal load is generally lower, making optimal sensitivity critical for reliable detection. Employing the cRE.21 for species level detection (CT = 0.4), we could detect *Aspergillus* species levels up to 4 MPM across all 20 simulated *Aspergillus* datasets of species included in the cRE.21 (Fig. 5b). When utilizing the dREM.260 (CT = 0.9) for genus level detection, detection of ≤4 MPM was achieved in 93% of the plasma and BAL ss-cfDNA simulations (n = 52), respectively (Fig. 5d). Surprisingly, the LoSD appeared relatively stable across different library sizes (Supplementary Fig. 10). Nevertheless, our findings discourage sequencing fewer than 40 million reads due to the adverse impact (i.e., substantial increase in the minimal required MPM) on the theoretical LoSD (Supplementary Fig. 10).

While the cRE.21 is thus established as the most sensitive for species level identification, we emphasize that the impact of database selection on the minimum required MPM can vary among different *Aspergillus* species. For example, the theoretical minimal *A. oryzae* MPM was 2 for cRE.21 and dREM.260, while for *A. niger* our analysis indicated a minimum of 0.25-0.5 MPM required with cRE.21 compared to 2 MPM with dREM.260 (Supplementary Fig. 9b-c). Exceptions notwithstanding, we confirmed our prior hypothesis that detection of *Aspergillus* species should be performed using cRE.21 supplemented by cREM.260 for genus level detection if species-specific results are negative.

### Diagnostic performance assessment: a proof-of-principle with seven IPA patients

As a proof-of-principle, we applied ss-cfDNA cfSPI to seven IPA cases which met the inclusion criteria (see *Methods*; Supplementary Fig 11; Table 1) and were classified as probable according to the EORTC/MSG criteria^9^ (A01-A05, A14-A15). Patient A03 had been classified as a probable IPA due to host factors (Table 1), repetitive high positive serum galactomannan, and suspected lesions on imaging (i.e., HRCT), but the initial BAL procedure showed negative results during diagnostic work-up. Subsequent repeat BAL procedure confirmed aspergillosis through positive BAL galactomannan at a later time point (this subsequent BAL sample was not included in our study). In our retrospective study, we subjected the initial BAL sample to ss-cfDNA NGS analysis. In total, we sequenced 7 BAL fluid samples (for A01-A05, A14-A15), paired with corresponding plasma (for A01-A05) and internal control plasma samples (for A01-A05) where possible.

**Table 1:** Clinical details of pediatric patients with probable IPA

	Patient information	Host factors	Radiology	Mycological evidence	Antifungal prophylaxis at time of diagnosis	EORTC/MSG definition
**A01**	Girl, 11, Post-HSCT for inborn error of metabolism. Persistent fever in the setting of severe GVHD. Antifungal therapy was started based on repetitive positive serum GM. Additional diagnostic testing was performed after acute dyspnea.	HSCT recipient Severe GVHD Prolonged corticosteroid use	Negative HRCT thorax	positive serum GM 3.58 Hyphae: yes Positive BAL GM 5.6 BAL culture: *A. fumigatus* Negative phenotypic/genotypic resistance test Functional endoscopic sinus surgery: negative culture, no hyphae Bronchoscopy: tracheobronchial pseudomembrane	Therapeutic L-AMB (daily 3 mg/kg)	Probable aspergillosis laryngo-tracheo-bronchitis
**A02**	Boy, 13, Post-HSCT for lymphoma Bronchiolitis obliterans treated with MP pulses. No clinical symptoms.	HSCT recipient Prolonged corticosteroid use	Suggestive HRCT thorax	Negative serum GM Hyphae: no Positive BAL GM 5.2 BAL culture: *A. fumigatus* Negative phenotypic/genotypic resistance test	No antifungal prophylaxis or antifungal therapy	Probable pulmonary aspergillosis
**A03**	Boy, 18 months, Post HSCT for HLH Persistent fever, pre-engraftment.	HSCT recipient Neutropenia	Suggestive HRCT thorax	Positive serum GM 6.9 Hyphae: no Negative BAL GM BAL culture: negative	Isavuconazole prophylaxis Adequate trough level	Probable pulmonary aspergillosis
**A04**	Boy, 5, with high-risk ALL. Induction phase Persistent fever.	Hematologic malignancy	Suggestive HRCT thorax	Negative serum GM Hyphae: no Negative BAL GM BAL culture: *A. fumigatus* Negative phenotypic/genotypic resistance test	Micafungin prophylaxis	Probable pulmonary aspergillosis
**A05**	Boy, 7, Post-HSCT for bone marrow failure Subfebrile temperature with coughing and tachypnea in the setting of graft failure.	HSCT recipient Neutropenia	Suggestive HRCT thorax	Negative serum GM Hyphae: no Negative BAL GM BAL culture: *A. fumigatus* TR34/L98H genotype resistance	Itraconazol prophylaxis No trough level measured	Probable pulmonary aspergillosis
**A14**	Girl, 1, with high-risk AML, induction therapy Persistent neutropenic fever	Hematologic malignancy Neutropenia	Suggestive HRCT thorax	Positive serum GM 2.5 Hyphae: no Positive BAL GM 7.2 BAL culture: negative	Micafungin prophylaxis	Probable pulmonary aspergillosis
**A15**	Boy, 16, Post-HSCT for NK cell malignancy Fever pre-engraftment	HSCT recipient Neutropenia	Suggestive HRCT thorax	Negative serum GM Hyphae: yes Positive BAL GM 9.7 BAL culture: *A. fumigatus* Negative phenotypic/genotypic resistance test	Micafungin prophylaxis	Probable pulmonary aspergillosis

*ALL* acute lymphoblastic leukemia, *HSCT* hematopoietic stem cell transplantation, *GVHD* graft versus host disease, *GM* galactomannan, *MP* methylprednisolone, *NK* natural killer.

**Fig. 6:**
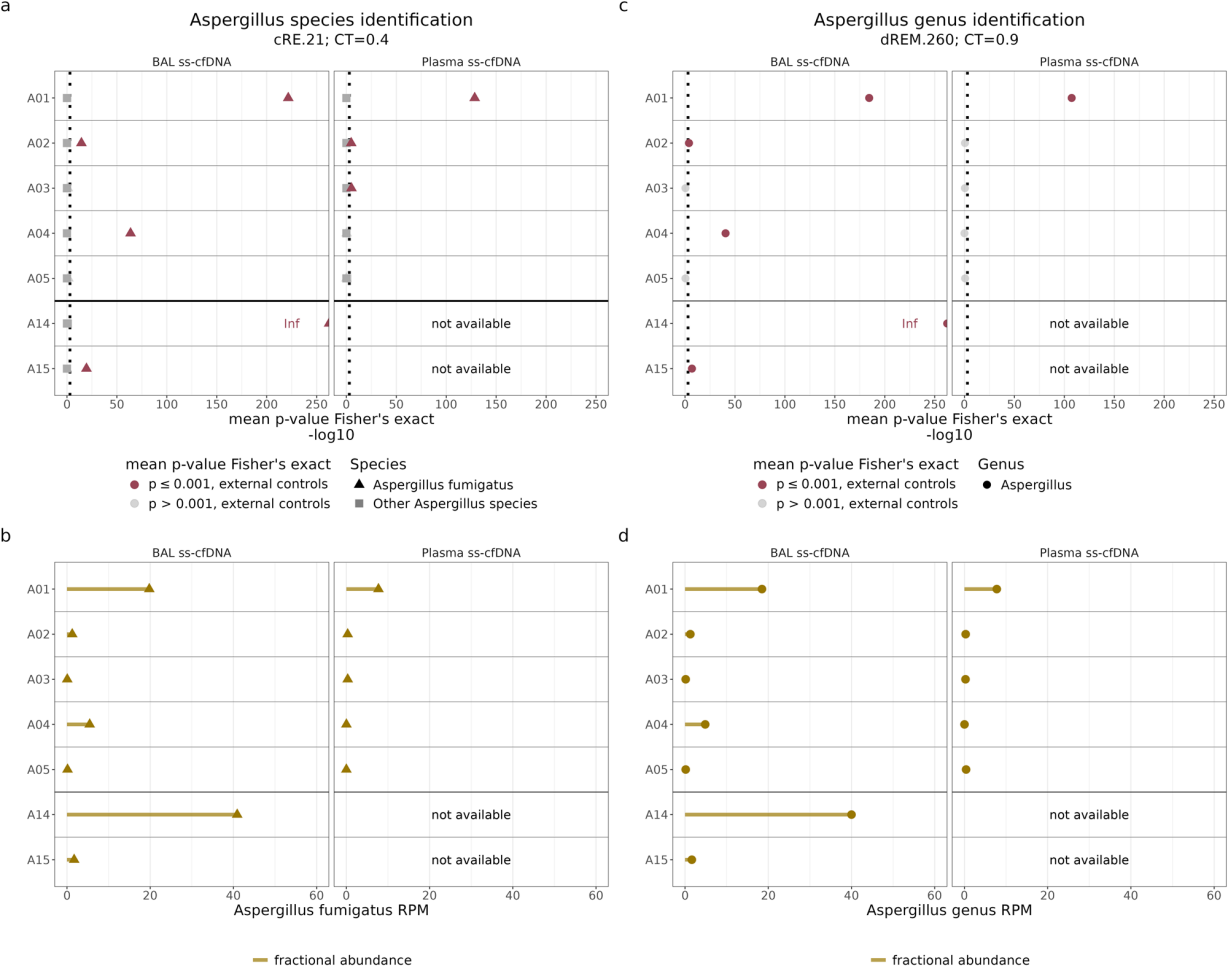
Elevated *Aspergillus* levels in a subset of IPA patient samples processed via ss-cfDNA NGS. To compare the fractional abundance of *Aspergillus* taxon in patient samples with invasive pulmonary aspergillosis (IPA) to external control pediatric cancer patient samples, the one-tailed Fisher’s exact test was utilized. **a**,**c**. This analysis was performed both **a**. at the species level, using the cRE.21 database (CT = 0.4), and **c**. at the genus level, using the dREM.260 (CT = 0.9) (see *Methods* for details). Dot plots display the mean -log10-transformed computed p-values, with the significance threshold set at p = 0.001 indicated by a vertical dotted line. Instances exceeding the significance threshold are highlighted in dark red. Lollipop plot displaying the fractional abundance of **b**. *A. fumigatus*, determined using the cRE.21 (CT = 0.4) and **d**. the fractional abundance of *Aspergillus* at the genus level, determined using the dREM.260 (CT = 0.9), in BAL and plasma IPA samples. Abbreviations: Inf, infinite value; not available, sample not available.

Comparing IPA liquid biopsy samples (plasma and BAL of A01-A05 and A14-A15, obtained at diagnosis) to 18 external control liquid biopsy samples from immunocompromised pediatric patients without suspected IPA infection (9 BAL; 9 plasma), *A. fumigatus* was significantly elevated in 5/7 of IPA BAL samples and 3/5 IPA plasma samples (Fig. 6a; mean pairwise Fisher’s exact test, p ≤ 0.001; for details see *Methods*). Importantly, none of the 18 external control samples tested positive with either of the databases (Supplementary 13b,d), indicating high specificity for ss-cfDNA NGS in immunocompromised pediatric patients. Similar results were observed when comparing IPA plasma to the internal control plasma as shown in Supplementary Fig. 12a (pairwise Fisher’s exact test, p ≤ 0.001). The positive samples exhibited fractional abundance of *A. fumigatus* ranging between 1.26 and 40.90 RPM in BAL and 0.31 and 7.71 RPM in plasma (Fig. 6b). In total, 6/7 patients (all except patient A05) had a positive result in at least one liquid biopsy using cfSPI ss-cfDNA NGS. Notably, cfSPI did not detect *A. fumigatus* in the BAL of patient A05, aligning with negative GM test results at the time of collection. Furthermore, cfSPI was the only molecular test that could detect *Aspergillus* in patient A03 compared to standard fungal molecular diagnostics (Table 1), showcasing the potential added value of our workflow.

The LoSD experiments demonstrated that if cRE.21-mediated species level diagnostics yield negative results, then the simultaneously conducted dREM.260-mediated genus level detection results should be interpreted. Among our IPA samples, the majority (8 out of 12) tested positive for *A. fumigatus* when using cRE.21, which led to no further interpretation of their dREM.260 results. The remaining 4 samples (BAL samples from patients A03 and A05, as well as plasma samples from patients A04 and A05), also tested negative with the dREM.260 database (Fig. 6c-d; and Supplementary Fig. 12b).

Notably, the internal control sample of patient A05 — the only patient in whom we could not detect *Aspergillus* with cfSPI in samples collected at diagnosis (Fig. 6; Supplementary Fig. 12) — showed elevated *A. fumigatus* cfDNA-levels 24 days before diagnostic work-up (Supplementary Fig. 13a; see Supplementary Fig. 11 for timeline). To gain insight in the time course of *Aspergillus* ss-cfDNA levels within this patient, we subjected two additional plasma samples (plasma A05-2 and A05-3; collected respectively 31 and 38 days prior to diagnosis) for sequencing. Both samples showed no elevated *Aspergillus* cfDNA levels (Supplementary Fig. 13a,c; employing the cRE.21 and dREM.260, resp.), suggesting a potential false positive *Aspergillus* observation in patient A05 obtained by cfSPI 24 days before diagnosis. However, the positive internal control sample for patient A05 could also represent the detection of a true biological signal detected by cfSPI, even though the GM test was negative. In conclusion, elevated cfDNA levels detected by cfSPI in the absence of clinical or radiological symptoms, as observed in patient A05, should be interpreted with considerable caution at this time.

As a quality control, we systematically aligned the reads classified as *A. fumigatus* from each sample to all *Aspergillus* genomes incorporated in constructing the cRE.21 database. Across positive cases, a median of 97.9% and 97.6% of *A. fumigatus* classified reads aligned with a high mapping quality to *A. fumigatus* strains A1163 ( = CBS121325) and Af293 (=CBS126847), respectively (Supplementary Fig. 14). Mapping to the closely related species *A. fischeri* and *A. novofumigatus*, on the other hand, only resulted in 54.0% and 36.2% mapped reads and low mapping quality (Supplementary Fig. 14). Minimal alignment was observed for all other *Aspergillus* species (Supplementary Fig. 14a). Reassuringly, a uniform distribution of cRE.21 classified reads was observed when mapping to the consensus genome *A. fumigatus* Af293 (Supplementary Fig. 15). Taken together, these findings confirm the presence of true-positive *A. fumigatus* classified cfDNA reads in our IPA patient liquid biopsy samples.

## Discussion

Our objective was to enhance the standardization of liquid-biopsy shotgun microbial NGS and to translate this knowledge into the cfSPI open-source analysis workflow that can be readily accessed and utilized by the scientific community. Our analysis involved both real-world (60 sequence libraries, generated from 46 different liquid biopsy samples; Supplementary Data 2) and simulated Illumina sequencing data (of 87 simulated datasets). We demonstrated that dual mapping to the human CHM13v2.0 and GRCh38.p14 references, along with the incorporation of both genomes into the classification database, is essential to prevent incorrect classification of human reads as microbial reads, potentially reducing the chances of false positive diagnoses. Additionally, we demonstrated that the NCBI RefSeq *kraken2* standard database is unable to detect most *Aspergillus* species, thus highlighting the necessity of database extension, particularly accomplished by incorporation of (pathogenic) fungal species and closely related taxa to enable species level *Aspergillus* detection. From a wet lab perspective, our results indicate that ss-cfDNA sequencing is a superior method for detecting fungal cfDNA compared to wcDNA sequencing. This is supported by the fact that our wcDNA isolation protocol yielded a maximum fungal count of only 2.55 RPM while the cfDNA yielded up to 12.55 RPM. The insights gained from our comparative research are poised to enhance the optimization of microbial NGS procedures and contribute to exploration of more precise and reliable liquid biopsy-based diagnostics for IMD.

In our retrospective proof-of-principle study, we demonstrated that cfSPI enables the detection of elevated levels of *A. fumigatus* in 71% (5/7) of BAL samples and 60% (3/5) of plasma samples compared to controls. This suggests that ss-cfDNA sequencing of BAL fluid supernatant — a relatively understudied liquid biopsy type in pediatrics — may offer diagnostic potential, given that BAL fluid fungal fractions typically exceed those in patient plasma samples. This finding aligns with previous research on detecting bacterial and viral pathogens in local body fluids^29,30^, as well as prior studies on *Aspergillus* detection in BAL samples from adults^31^. Nevertheless, due to the minimally invasive nature of plasma sampling, there remains a strong interest in exploring the diagnostic potential of plasma microbial NGS for IMD. The observed sensitivity of plasma sequencing (60%) is consistent with previous sequencing findings in adult cohorts, where sensitivity is ranging between 38.5%^16^ and 61%^15^, suggesting that BAL testing might be unnecessary in up to 60% of IPA cases. Although this is a small proof-of-principle cohort, sequencing of BAL samples resulted in a better sensitivity than the standard diagnostics in BAL^10,11^. Importantly, we noted minimal false positive rates, indicating that cfSPI offers compelling evidence for the presence of *Aspergillus*.

While the exclusive detection of *A. fumigatus* in our IPA patient set is in line with findings by Hill et al. ^15^, the size and composition of our IPA patient set limits our ability to evaluate the effectiveness of cfSPI ss-cfDNA NGS in identifying other *Aspergillus* species. Nonetheless, our simulations suggest the capability of detecting diverse *Aspergillus* species and distinguishing clinically relevant ones such as *A. fumigatus* and *A. terreus*. The limited availability of retrospective samples also restricts our ability to definitively demonstrate (or rule out) potential additional benefits of dREM.260 as a supplementary test for pan-*Aspergillus* detection at the genus level. Therefore, our hypothesis that in cases where the pathogen in a patient is not covered by the compact curated cRE.21 database, resulting in negative species-specific findings, the supplementary dREM.260 results could be crucial for comprehensive pan-*Aspergillus* detection, requires further investigation and validation. Moreover, a comparison between our optimized ss-cfDNA NGS workflow for *Aspergillus* detection and conventional diagnostics remains impossible due to the highly limited size of our proof-of-principle and exclusive application of ss-cfDNA to probable IPA cases, thus precluding insight into its diagnostic performance in fungal infections.

To advance this technical research towards potential clinical implementation, our workflow still requires an external validation cohort comprising pediatric patients suspected of IMD, including those classified as possible, probable, and proven cases. A critical step in demonstrating clinical applicability is testing whether our cfSPI workflow maintains its sensitivity and specificity outside of a retrospective, controlled research environment. By making our workflow open-source and designed for in-house use, we aim to facilitate external validation efforts while minimizing turnaround times by eliminating the need for sample shipping. This approach also supports the development of local expertise and ensures adaptability to specific populations through the seamless integration of customized classification databases. These features provide a significant advantage over commercial tests like the Karius Test, of which the database notably shares substantial overlap with the genomes integrated in our cRE.21 database (see Supplementary Fig. 16).

Collectively, our study provides valuable insights in the use of ss-cfDNA NGS for *Aspergillus* detection in pediatric immunocompromised hosts. The cfSPI framework introduced here has the potential to expedite future fungal-NGS investigations through its open-source analysis workflow. Our findings, complemented with new simulations and LoSD analysis and coupled to benchmarking using short-read sequencing data from fungal culture isolates, can solidify the groundwork for enhancing these open-source detection tests, not only for *Aspergillus* but also for other pathogens responsible for IMD.

## Methods

### Aim of this study

The aim of this study is optimization of the microbial NGS workflow tailored to *Aspergillus* detection in liquid biopsy samples. This involves shotgun sequencing of liquid biopsy samples, such as blood or BAL fluid, with the objective of sequencing intact microbial wcDNA or small DNA molecules from degraded microbes (i.e., cell-free DNA, cfDNA). Following microbial NGS, taxonomic identification of the DNA source is performed by tracing the origin of sequenced DNA molecules. This process includes human read subtraction, taxonomic classification, and subsequent statistical analysis to determine if there is supporting evidence for a pathogenic microbe. In this study, we refined both the wet-lab and computational workflow (cfSPI) by optimizing six key steps (Q#1-6) in microbial NGS-based fungal diagnostics, as detailed in Fig. 1.

### IPA patients

#### Diagnostic work-up of IMD suspected patients includes clinical microbiology

At the Princess Máxima Center for Pediatric Oncology (PMC) in Utrecht, The Netherlands, patients suspected of IMD are evaluated by a chest high-resolution computerized tomography (HRCT) scan. If suspected lesions for IMD are seen on HRCT, a BAL is performed. The BAL fluid provides material for microscopy, fungal culture, galactomannan (GM) assay (Platelia Aspergillus Ag, Bio-Rad), and molecular diagnostics. A BAL GM > 1.0 is considered to be positive according to the criteria of the European Organization for Research and Treatment of Cancer and Mycoses Study Group (EORTC/MSG) criteria^9^.

#### Retrospective proof-of-principle: inclusion of IPA patient samples

We included plasma and BAL fluid from immunocompromised pediatric patients who were diagnosed with an IPA at the PMC between 2020 and 2023. We searched manually for cases with probable or proven IPA according to the EORTC/MSG criteria^9^ (Table 1).

The date of diagnosis was based on the date of BAL retrieval with a positive microbiological finding. One BAL fluid sample and one blood plasma sample (time-matching the BAL fluid sample or at least one or two days around the date of diagnosis) were used from each case, as well as a plasma sample collected approximately fourteen days prior to the date of diagnosis (Supplementary Fig. 11). These plasma samples collected earlier in time served as internal control samples. We also included BAL fluid and plasma samples from pediatric immunocompromised patients without IPA, so called external control samples. BAL fluid control samples were used from patients pre-HSCT, during an anesthetic procedure for line insertion^32^. All materials used for this study were clinical samples routinely stored at -70°C. Before freezing, the plasma samples were prepared by centrifuging EDTA plasma to remove the cells. BAL fluids were stored directly after use without prior centrifugation. Our study sets a high standard by incorporating both internal and external control samples, surpassing other studies that solely reported sequencing results from suspected IPA cases.

In total, this study encompassed 25 pediatric patients, including 7 diagnosed with probable IPA and 18 external immunocompromised controls (9 plasma samples and 9 BAL fluids). All included patients provided written informed consent for participation in the biobank for storage and use of their rest materials (International Clinical Trials Registry Platform: NL7744; https://onderzoekmetmensen.nl/en/trial/21619). For use of samples and data in this study we refer to local Biobank and Data Access Committee approval (approval number PMCLAB2022.364). All patients have also provided informed consent for sequencing and use of these data for publication. This study was conducted in compliance with the principles of the Declaration of Helsinki.

#### Diagnostic sensitivity and specificity

One of the aims of this pilot study was to examine the diagnostic sensitivity of microbial NGS-based detection of *Aspergillus* DNA in immunocompromised pediatric patients. The diagnostic sensitivity was gauged by the proportion of probable IPA patients displaying significantly heightened levels of at least one *Aspergillus* species, or at the *Aspergillus* genus level, in at least one liquid biopsy sample (blood or BAL fluid) in comparison to external controls.

The specificity of microbial NGS was determined by the proportion of external control patient samples that displayed significantly elevated levels of at least one *Aspergillus* species or at the *Aspergillus* genus level, in at least one liquid biopsy sample (blood or BAL fluid) in comparison to all other external controls.

### Wet-lab

#### Sample preparation and DNA isolation

Plasma was obtained from EDTA blood samples by centrifugation, 10 min at 1500 g, followed by an additional centrifugation step for 5 min at 12,000 g, to remove all cells (both centrifugation steps at room temperature). Plasma was subsequently stored at -80 *°*C. Fresh BAL fluid samples were stored at -80 *°*C until further processing. To separate the (whole) cells from the BAL fluid the samples were centrifuged for 5 min at 13,000 rpm at 4 °C. Subsequently, the pellet and supernatant were processed in separate DNA isolation methods for wcDNA sequencing and cfDNA sequencing, respectively.

cfDNA nucleic acids were isolated from BAL supernatant, EDTA plasma and sterile Dulbecco’s Phosphate Buffered Saline (DPBS), using the Circulating Nucleic Acid Kit (Qiagen, 55114) with the following modifications to the manufacturer’s protocol: A select set of our BAL fluid and plasma samples were complemented with DPBS, up to a total volume of 2 mL. Furthermore, the lysis time was increased from 30 to 60 minutes and the final elution of cfDNA was done with Nuclease Free water (Invitrogen, 10977-035).

DNA from 5 (out of 13) BAL pellets was extracted from whole cells by mechanical bead beating (see Supplementary Data 2, “internal” DNA isolation) where 50 µL DPBS and a 5 mm stainless steel-bead (Qiagen, 69989) were added to the BAL pellet. Samples were beaten for 2 min (with a frequency of 2 5 1/s) on the TissueLyser II (Qiagen) and subsequently diluted in ATL buffer (Qiagen, 939011) and transferred to a fresh tube. Additional ATL buffer was added to a total volume of 300 µL, with an overnight incubation at 56°C after addition of Proteinase K (Qiagen, 19131). wcDNA isolation was performed using the DNeasy Blood & Tissue Kit (Qiagen, 69506), following manufacturers protocol adjusting subsequent volumes to accommodate the larger lysis volume.

From the remaining 8 of our 13 BAL pellet samples (see Supplementary Data 2, *external* DNA isolation) wcDNA was isolated according to a standard protocol for fungal DNA isolation prior to RT PCR (UMC Utrecht, Dept. Medical Microbiology, The Netherlands). In short, BAL pellets were bead beaten and snap lysed (by freezing them at -80 °C and heating them to 96 °C). After addition of a lysis buffer, nucleic acids were isolated using the MagNA Pure system (Roche).

All DNA samples were quantified using the Qubit dsDNA High Sensitivity assay kit or Broad Range assay kit (Thermofisher Scientific, Q32854 and Q32853, respectively). The DNA fragment length distribution and concentration of the cfDNA was evaluated using the TapeStation 2200, D1000HS kit (Agilent, 5067-5585).

#### Preparation of next-generation sequencing libraries

For ss-ligation based DNA-capture the SRSLY PicoPlus NGS Library Prep Kit for Illumina (Claret Bioscience, CBS-K250B-96) was used, according to the manufacturer’s Moderate Fragment Retention version of the protocol, using max 5 ng cfDNA as input and 11 PCR cycles. For ds-ligation-based cf/wcDNA-capture the KAPA Library Preparation Kit (Roche) was used, with a maximum of 50 ng of input DNA and 8-12 PCR cycles. For ds-capture of the BAL pellet DNA, the manufacturer’s protocol was followed. For ds-capture of cfDNA the following changes to the manufacturer’s protocol have been made: no fragmentation step and following similar bead clean up steps as the moderate fragment retention protocol to improve the yield of small fragments. All library preparations were quantified using the Qubit dsDNA High Sensitivity Assay Kit (Thermofisher Scientific, 32854) and size distribution was analyzed using the TapeStation 2200, either the D1000 and/or D5000 kits (Agilent, 5067-5583/5067-5589). Some samples showed an aberrant size distribution (substantial fraction of >700 bp fragments); these samples were subjected to a bead-based size selection protocol (see Supplementary Data 2), to remove long fragments as Illumina sequencing is optimized for fragments <700 bp. Concentration (see Supplementary Data 2, total DNA yield in ng) and size were re-evaluated after size selection. Samples were pooled equimolarly and submitted for sequencing.

### Next-generation sequencing

Sequencing of all 60 Illumina libraries was conducted on the NovaSeq 6000, 2×150 bp reads, resulting in 42 to 218 million reads per ss-cfDNA sample, 37 to 126 million reads per ds-cfDNA patient sample, and 34 to 62 million reads per wcDNA patient sample (see Supplementary Data 2).

### Read simulations

In order to evaluate the impact of kraken2’s reference hash-table database composition and confidence threshold on the *Aspergillus* classification accuracy, we simulated cfDNA-like datasets from 87 complete, scaffold, or draft genomes derived from the NCBI RefSeq, among which 55 *Aspergillus*, 7 *Penicillium*, and 25 other pathogenic fungal genomes with a Illumina read simulator tool named ReSeq^33^. To create a realistic error profile of sequencing reads, the unprocessed ss-cfDNA sequencing reads from plasma of patient A01 (i.e., A01Pasp, for details on sample workup see Supplementary Data 2) sequenced with Illumina Novaseq 6000 2×150 bp were mapped to the human GRCh38.p14 reference genome using Bowtie2 (run with option “-X 2000”) alignment software^34^. Subsequently, the reference sequence statistics were determined using ReSeq (option “--statsOnly”), and 151 bp paired-end reads were simulated *in-silico* using the illuminaPE ReSeq command (option “--noBias”)^33^. For each genome we simulated between 99,050 and 101,023 reads; for details on simulated datasets and the number of reads per dataset see Supplementary Data 1.

### Database construction

For the purpose of fungal nucleic acid detection, we utilized 9 kraken2 classification databases, namely uR.7, uR.7 w/o CHM13v2, cRE.21, dRE.21, uRE.21, uRE.31, dRE.31, dREM.258 and dREM.260 of which details on the construction and composition are reported in Supplementary Data 5.

Prior to hash-table construction, specific genomic regions of the reference genomes were masked to prevent spurious misclassifications. This masking procedure involved *dustmasking*, where low-complexity regions were masked using the DUST algorithm^21^ as advised by the developers of kraken2, more rigorous *decontamination* efforts thereby masking contaminant organism sequences, or a combination of dustmasking and decontamination (i.e., full *cleanup*), such as in the work of Lu and Salzberg^35^. Contaminant organisms’ sequences are sequences within genome assemblies that do not accurately represent the organism’s genetic information.

Database names indicate the masking procedure used (**u**naltered, **d**ustmasked or **c**leaned), the database sources (**R**efSeq, **E**uPathDB and/or **M**ycoCosm) and the number of *Aspergillus* species included (Supplementary Fig. 2). For example, **cRE.21** refers to the **c**leaned version of a combination of **R**efSeq and **E**uPathDB with a total of **21** *Aspergillus* species. Each database was built using the NCBI taxonomic information, which was downloaded on 05-05-2023.

The NCBI RefSeq^22^ is a comprehensive and curated collection of nucleotide sequences, encompassing a wide range of species, which — in the context of kraken2 classification — are often used as a standard reference hash-table database construction. Using the “kraken2-build --download-taxonomy*”* command, genomes of the NCBI RefSeq were downloaded on 15-05-2023 (RefSeq release 218, file creation date 05-05-2023), including 1,495 archaea, 285,827 bacteria, 498 fungi, 98 protozoa, 14,979 viral and 1 human sequence plus 3,137 contigs which were part of the UniVec_Core. UniVec_Core comprises oligonucleotide and vector sequences sourced from bacteria, phage, yeast, and synthetic constructs, excluding vector sequences of mammalian origin.

The EuPathDB encompasses a curated set of genomic sequences of 386 pathogenic fungi, protists, oomycetes as well as evolutionarily related non-pathogenic species^26^. EuPathDB genomic sequences were downloaded on 16-05-2023 (file creation date was 28-10-2020), consisting of the following subsets: AmoebaDB (n = 30), CryptoDB (n = 18), FungiDB (n = 164), GiardiaDB (n = 10), MicrosporidiaDB (n = 35), PiroplasmaDB (n = 10), PlasmoDB (n = 45), ToxoDB (n = 33), TrichDB (n = 1) and TriTrypDB (n = 42). Upon inspection post-download, we observed that two fungal genomes were omitted from the purified edition of FungiDB-46. Jennifer Lu, one of the authors of the contaminant removal paper^35^, kindly supplied us with the latest version of these two genomes:

*FungiDB-54_PgraminisCRL75-36-700-3_Genome_cleaned_v_final.fna* and *FungiDB-54_Ptriticina1-1BBBDRace1_Genome_cleaned_v_final.fna*.

An updated version of the *seqid2taxid.map* used for database construction was also provided by Jennifer Lu. In total, EuPathDB version 46 includes 27 *Aspergillus* genomes, representing 21 *Aspergillus* species, while version 64 contains 38 genomes, representing 31 *Aspergillus* species.

The MycoCosm^27^ is a web-based resource and information portal developed by the Joint Genome Institute (JGI) for fungal genomics. It provides access to a comprehensive collection of fungal genomes, associated functional annotations, and tools for comparative analysis. We acquired all 763 genomic sequences of *Aspergillus* assembly scaffolds from this resource, along with their corresponding taxonomic annotations.

### Host read subtraction

Our objective was to eliminate potential false positives microbial reads originating from incomplete host read subtraction. To achieve this, we employed mapping to either the GRCh38.p14, CHM13v2, or a dual-mapping utilizing a consolidated reference index for Bowtie2 alignment, encompassing both GRCh38.p14 and CHM13v2, thereby avoiding a two-step mapping process.

### Sequencing and synthetic data processing: the cfSPI-pipeline

Illumina sequencing and synthetic data were processed using the Snakemake^36^ cfSPI-pipeline available in our Github repository (https://github.com/AEWesdorp/cfSPI/cfspi/). In short, duplicates were removed (using nubeam^37^), after which high-quality sequencing data was generated (using fastp^38^) by default removal of low quality reads and usage of a low complexity filter as well as by adapter removal and removal of short (<35 bp) reads (using AdapterRemoval^39^). After subtraction of host sequences by mapping to the human reference genome using Bowtie2^34^, the remaining paired-end reads were taxonomically classified using kraken2^20^ with the 9 kraken2 databases specified above, employing a confidence threshold (CT) ranging from 0.0 (no filter on the fraction of *k-mers* matching, default setting) to 1.0 (100% of *k*-mers within the read match a taxa, very stringent setting), increments of 0.1.

### Remapping of classified reads

Reads classified as *Aspergillus* at the species level with the cRE.21 database, along with all lower-ranking taxa within the same clade, were aligned to the respective *Aspergillus* species genomic sequences used for the cRE.21 database construction, through Bowtie2.

### Relative abundance per taxon

The relative abundance per taxon within each sample was quantified as Reads Per Million (RPM), a normalized measure that accounts for i.e., differences in sequencing depth. RPM was calculated according to the following formula:

1 $${RPM}={totalSumTaxonReads}/{totalNumQCReads}* {1,000,000}$$

*totalSumTaxonReads* corresponds to the number of reads classified at each taxon (e.g., genus- or species level) and all lower-ranking taxons belonging to the same clade. *totalNumQCReads* is the count of reads that passed quality control (i.e., number of reads remained after duplicate removal, low-quality and low-complexity reads filtering, and adapter removal; see Supplementary Fig. 1a).

### Limits of Significant Detection

In order to explore the relationship between fungal background levels in immunocompromised individuals, the observed classification rate in simulated datasets, and the resulting theoretical ‘Limits of Significant Detection’, we formulated the following methodology. First, we defined our taxon, database, and CT of interest. Second, we set the *theoretical sequencing depth* (*tSD*), ranging from 10 to 100 million reads (with intervals of 15 million) as well as a theoretical number of *molecules per million* (*mpm*) ranging from 0.25 to 4096. Third, we obtained the median *background abundance* (*ba*) observed in our external immunocompromised samples for the specified taxon, database, and CT of interest (normalized, RPM). Fourth, we determined the *classification rate* (*cr*) observed in our simulated datasets for the specified taxon, database, and CT of interest. Subsequently, we applied the following calculation to determine the total taxon read count in our artificial sample, rounded to the nearest integer:

2 $${total\; taxon\; count}=\left\lfloor ({tSD}* {ba})+({tSD}* {mpm}* {cr})+0.5\right\rfloor$$

Following this, we applied a one-tailed Fisher’s exact test to assess whether the observed total taxon count in our theoretical samples significantly differed from that in our external control samples. A mean p ≤ 0.001 was deemed statistically significant. The lowest mpm value with a p-value ≤ 0.001 was reported as the MPM.

### Identification of elevated Aspergillus levels

Following taxonomic classification of all clinical samples, we conducted a one-tailed Fisher’s exact test to assess statistical differences in the read count of a specified taxon between our patient samples and internal/external control samples. This test was based on comparing the following two counts in the contingency tables:

1. The number of reads classified at the taxon of interest, including reads at the specified taxonomic level (e.g., genus- or species level) and all lower-ranking taxa within the same clade.
2. The number of reads remaining after duplicate removal, low-quality, and low-complexity read filtering, excluding those classified at the taxon of interest.

The significance level was set at p ≤ 0.001, calculated by deriving the mean of all Fisher’s exact tests conducted across the samples. This analysis aimed to identify meaningful differences in taxon-specific read counts between patient and control groups, considering the overall composition and diversity of microbial taxa in the studied samples.

### Statistical analysis

To evaluate the influence of computational choices, including host-read subtraction and database composition, we utilized the one-tailed paired t-test. For comparisons involving sample types and library preparation, we applied the one-tailed Wilcoxon rank-test. To account for multiple testing, we employed Bonferroni correction in these analyses.

### Software

Data and statistical analyses were conducted in R (v.4.2.0). Figures were generated in R (v.4.2.0), and illustrations created using BioRender and Adobe Illustrator (2024, v28.6).

## Supplementary information

Supplementary Information

Supplementary Data

## Data Availability

Data will be made available on reasonable request, through the European Genome-phenome Archive (EGA) under accession number EGAS00001008021. Additionally, taxonomic abundance matrixes are provided via the GitHub repository upon publication.

## Code availability

Details regarding the cfSPI pipeline can be found here: https://github.com/AEWesdorp/cfSPI/cfspi/. The code, encompassing data simulation scripts and the analysis of data presented in this manuscript, are available here: https://github.com/AEWesdorp/cfSPI/.
