# Supplementary material for: NGS-based Aspergillus detection in plasma and lung lavage of children with invasive pulmonary aspergillosis
Source: NPJ Genom Med. 2025 Mar 17;10:24. doi: 10.1038/s41525-025-00482-8 (PMC11914610; doi:10.1038/s41525-025-00482-8)
Supplement: Supplementary file 1 — Supplementary Information [file 41525_2025_482_MOESM1_ESM.pdf]

Supplementary Material for
NGS-based *Aspergillus* detection in plasma and lung lavage of children with invasive pulmonary aspergillosis

Emmy Wesdorp[1,2], Laura Rotte[3,*], Li-Ting Chen[1,2,*], Myrthe Jager[1,2,*], Nicolle Besselink[1,2], Carlo Vermeulen[1,2], Ferry Hagen[4,5,6], Tjomme van der Bruggen[7], Caroline Lindemans[3,8], Tom Wolfs[8], Louis Bont[8,#], Jeroen de Ridder[1,2,#]

[1] Center for Molecular Medicine, University Medical Center Utrecht, Utrecht, The Netherlands; [2] Oncode Institute, Utrecht, The Netherlands; [3] Hematopoietic stem cell transplantation, Princess Máxima Center for Pediatric Oncology, Utrecht, The Netherlands; [4] Westerdijk Fungal Biodiversity Institute, Utrecht, The Netherlands; [5] Department of Medical Microbiology, University Medical Center Utrecht, Utrecht, The Netherlands; [6] Institute for Biodiversity and Ecosystem Dynamics, University of Amsterdam, The Netherlands; [7] Department of Medical Microbiology, University Medical Centre Utrecht, The Netherlands; [8] Department of Pediatric Infectious Diseases and Immunology, Wilhelmina Children's hospital, UMC Utrecht, Utrecht, The Netherlands.

Supplementary Data 1-5

Supplementary Figure 1-16

Supplementary Data 1. Number of reads in simulated cfDNA-like Illumina sequencing datasets.
This table provides the number of paired-end cfDNA-like Illumina reads simulated per strain.

Supplementary Data 2. Overview of BAL and plasma samples, including sample workup details and Illumina read count.
This table outlines the details of the sample workup and includes information on Illumina read counts for both BAL (bronchoalveolar lavage) and plasma samples. IPA: invasive pulmonary aspergillosis; BAL: bronchoalveolar lavage; ss-cfDNA: single-stranded library preparation of cfDNA (using SRLSY kit); ds-cfDNA: single-stranded library preparation of cfDNA (using KAPA kit); ds-wcDNA: double-stranded library preparation of whole-cell DNA (using KAPA kit); BB-SS: bead-based size selection.

Supplementary Data 3. Overview of kraken2 reference hash-table databases and the Aspergillus species they contain.
This tavle shows which *Aspergillus* species are included in different kraken2 reference hash-table databases. A value of 0 means the species is absent, and a value of 1 means it is included.

Supplementary Data 4. Overview of strains used to simulate cfDNA-like Illumina sequencing datasets and their species' inclusion in kraken2 reference hash-table databases.
An overview of the strains used to simulate cfDNA-like data and their species' presence in kraken2 reference hash-table databases. A value of 0 means the species is not included in the database, while a value of 1 means the species is included.

Supplementary Data 5. Details on the construction of kraken2 reference hash-table databases.
Outlines the process for constructing kraken2 reference hash-table databases.

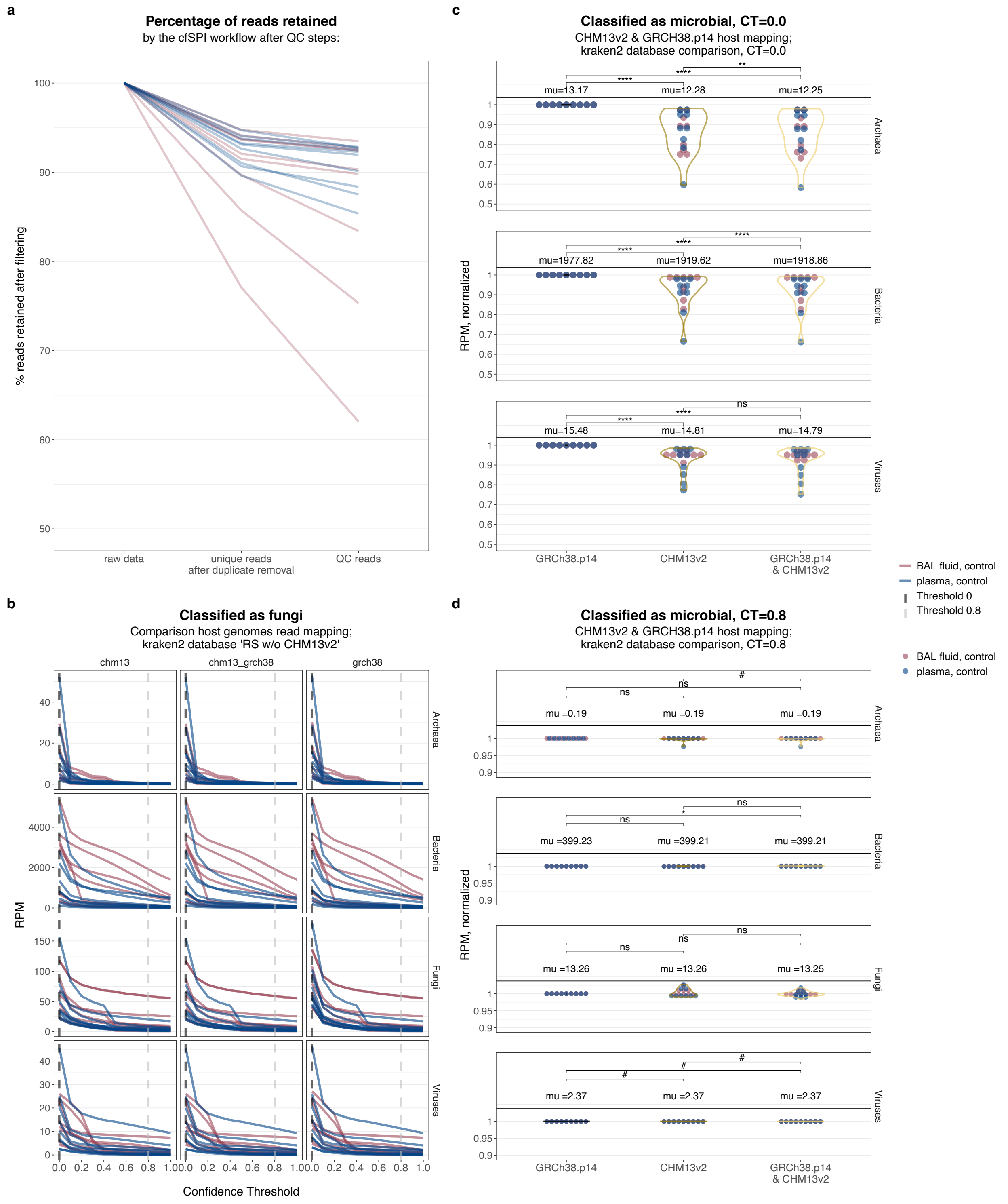

Supplementary Figure 1. Impact of CHM13v2 host genome mapping on archeal and viral read quantification

a. Percentage of quality controlled (QC) reads retained by the cfSPI workflow after 1) removal of duplicates and 2) removal of low-quality and short reads. **b.** CT dependent fraction of archaeal, bacterial, fungal and viral classified reads, after host read subtraction by reference genome mapping. Black and grey lines represent CT of 0.0 and 0.8, respectively. **c-d.** Fractional abundance of archaeal, bacterial, fungal (only in **d.**) and viral kraken2 classified reads when utilizing a **c.** CT of 0.0 (kraken2's default) or a **d.** CT of 0.8 (for a high precision [20]), after subtracting host reads via reference genome mapping, normalized to the old version of the human genome assembly (GRCh38.p14). In **a-d.**, each data point/line represents one

control sample (9 BAL; 9 plasma), with colors indicating the sample type. In **c-d.**, Mean PRM values are denoted as 'mu'. Statistical analysis included one-tailed paired t-tests with Bonferroni correction (*, $p \leq 0.05$; **, $p \leq 0.01$; ****, $p \leq 0.0001$; ns, $p > 0.05$; #, t-test not performed due to all paired values being identical), utilizing the fractional abundance in RPM.

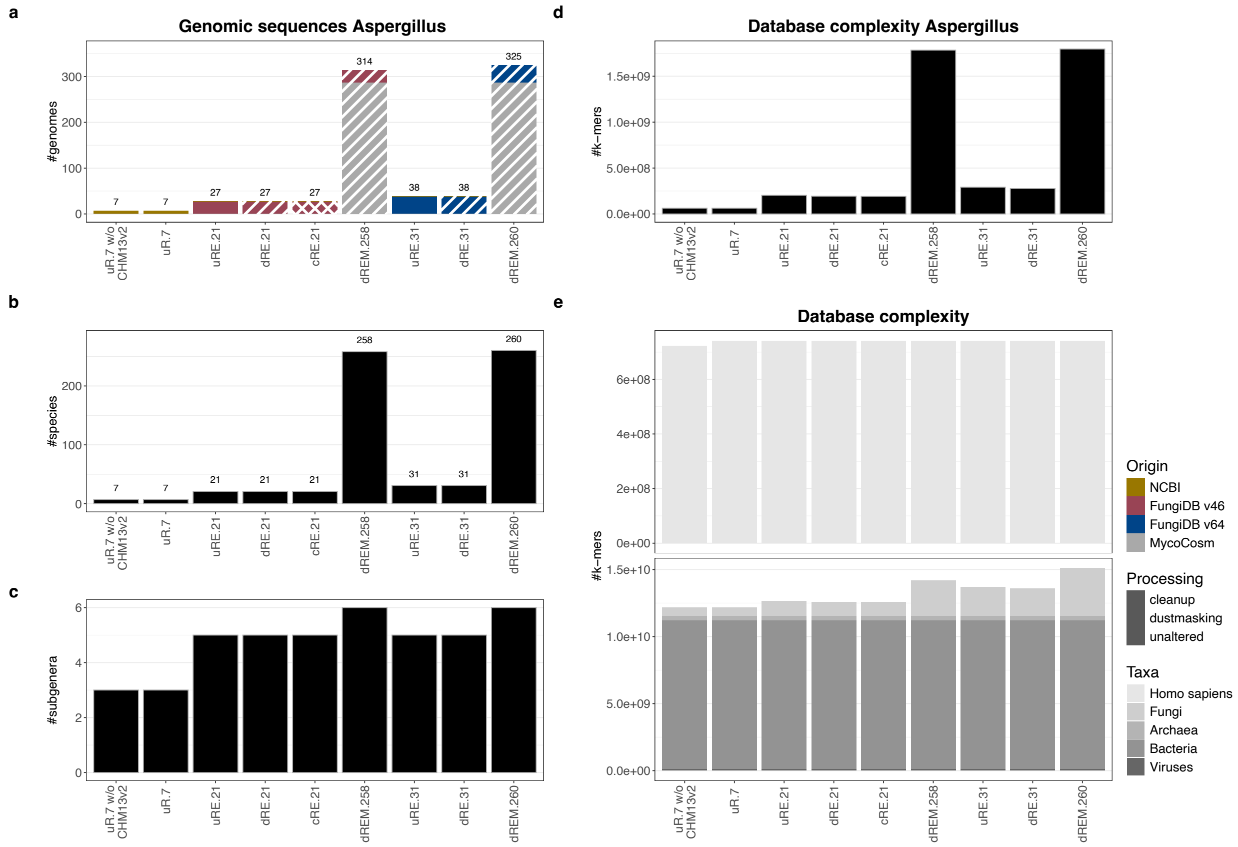

Supplementary Figure 2. Comparison between kraken2 classification database complexity

a. Number of *Aspergillus* genomes, sourced from NCBI RefSeq, FungiDB (a subset of the EuPathDB), and/or MycoCosm, within diverse kraken2 classification databases (x-axis). Genomic sequences underwent various processing steps, including cleanup (crossing lines), dustmasking (diagonal lines), or no processing (denoted as 'u' for unaltered), before database construction. The number of **b.** *Aspergillus* species and **c.** *Aspergillus* subgenera in the diverse databases. **d.** Count of *Aspergillus* *k*-mers in diverse hash-table databases. **e.** Count of human and microbial *k*-mers in diverse hash-table databases. Database characteristics are summarized on the x-axis using colors to reflect the origin of the *Aspergillus* genomic sequences, patterns to denote the processing status of the *Aspergillus* genomes in the database.

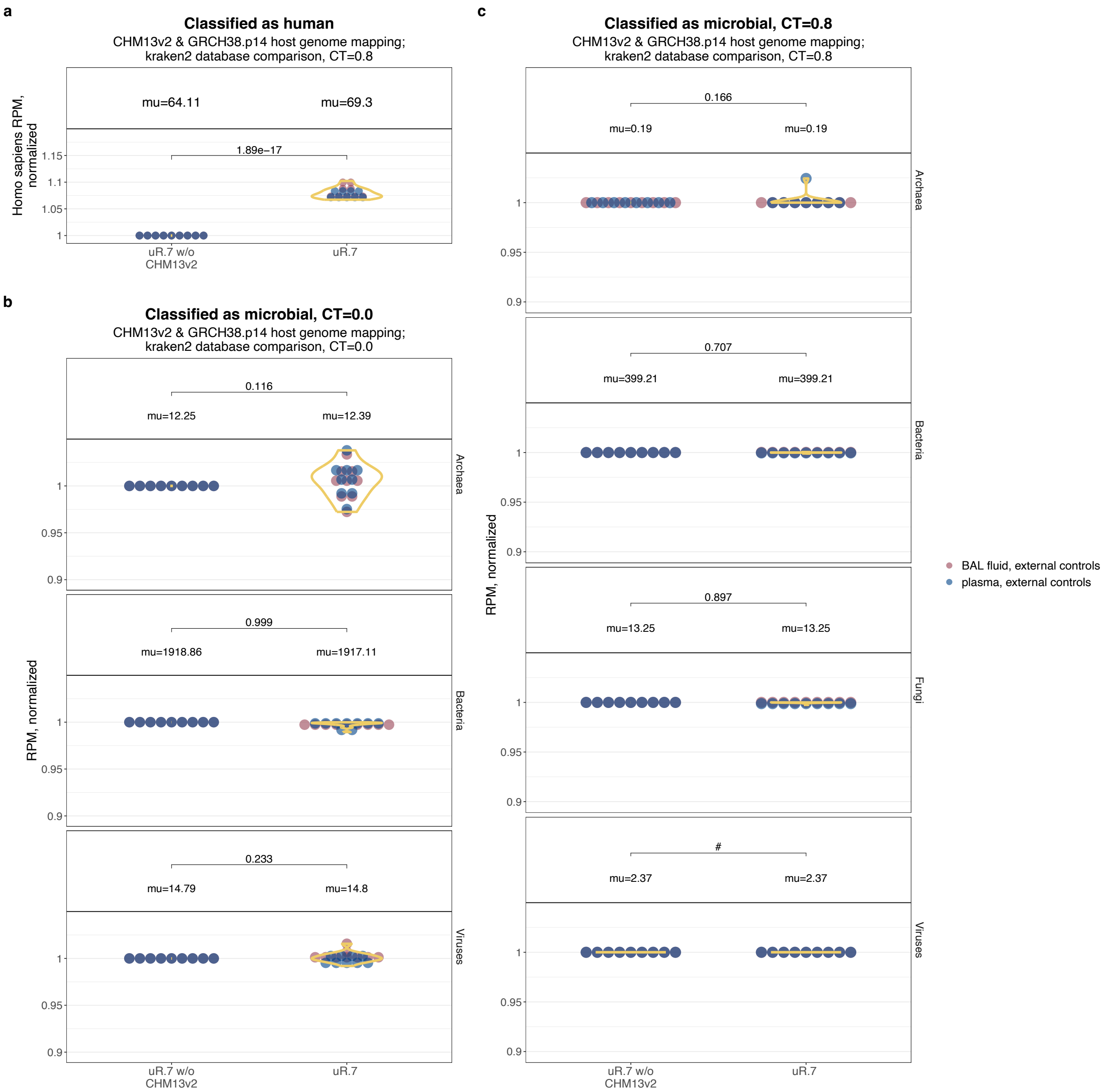

Supplementary Figure 3. Integration CHM13v2 into the kraken2 database: exploring implications for host and microbial read counts

a. Fractional abundance of human classified reads when utilizing the 'CHM13v2-containing uR.7' or 'uR.7' database for kraken2 taxonomic classification (CT=0.8, kraken2's default) after dual-mapping to the host genome, normalized to 'uR.7'. **b-c.** Fractional abundance of archaeal, bacterial, fungal (only in **c.**) and viral kraken2 classified reads when utilizing the 'CHM13v2-containing uR.7' or 'uR.7' database for kraken2 taxonomic classification with a CT of 0.0 and 0.8, resp., after dual-mapping to the host genome, normalized to 'uR.7'. Each data point represents one control sample (9 BAL; 9 plasma), with colors indicating the sample type. Mean RPM values are denoted as 'mu'. Statistical analysis included one-tailed paired t-tests with Bonferroni correction (#, t-test not performed due to all paired values being identical), utilizing the fractional abundance in RPM.

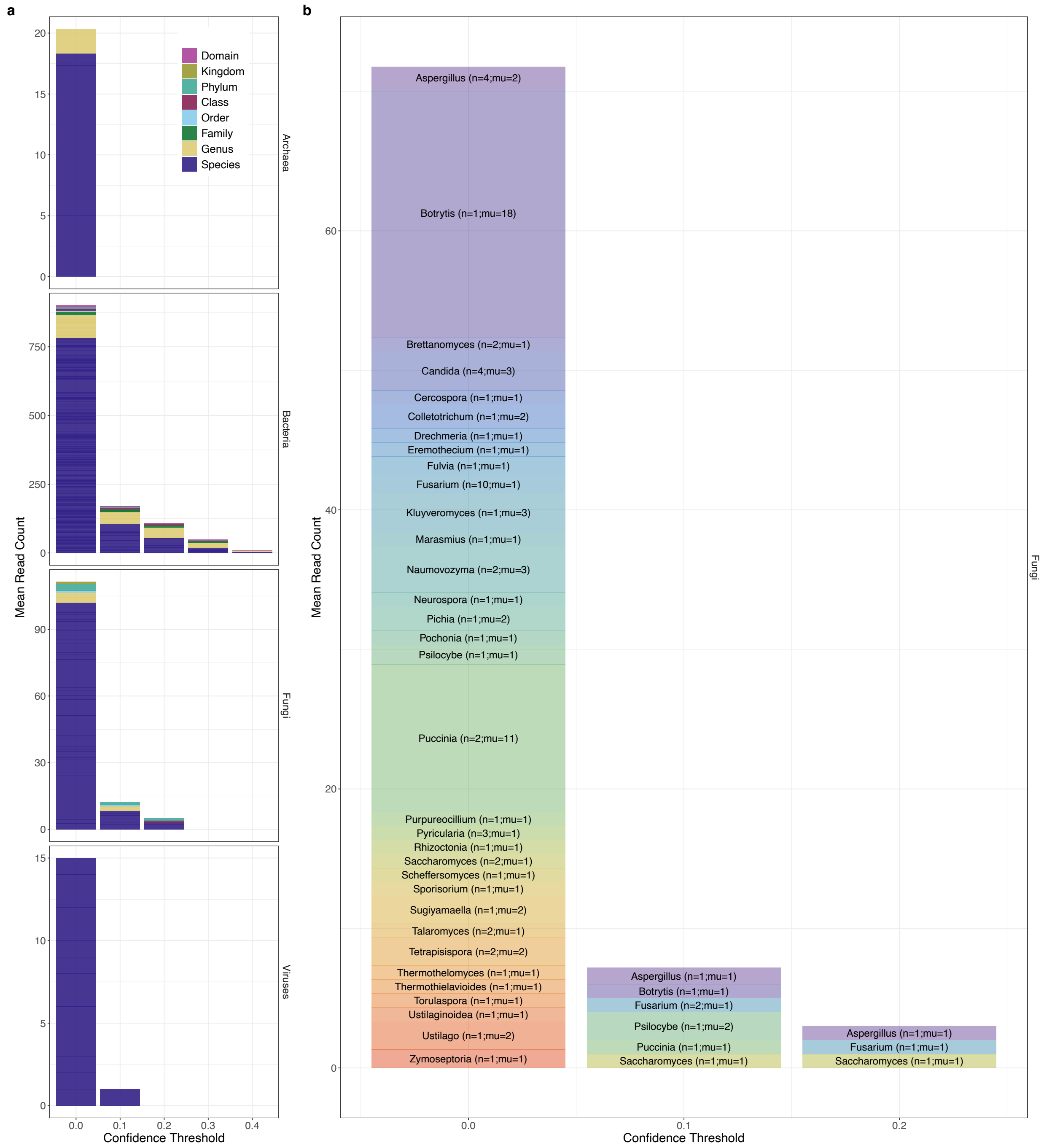

Supplementary Figure 4. Exclusion CHM13v2 in the kraken2 database leads to misclassification of human reads as fungal species

We traced individual reads classified as human by the 'CHM13v2-containing uR.7' and assess their classification in the 'uR.7 w/o CHM13v2' database to identify misclassification. **a.** Mean classified microbial (i.e. archaeal, bacterial, fungal and viral) read counts in control samples (n=18) at various CTs. Colors indicate the microbial taxonomic level at which reads were classified. **b.** Mean counts of fungal species level classifications in control samples (n=18). Colors represent fungal genera, with numerical annotations denoting the count of fungal species within each genus and the mean number of reads ('mu').

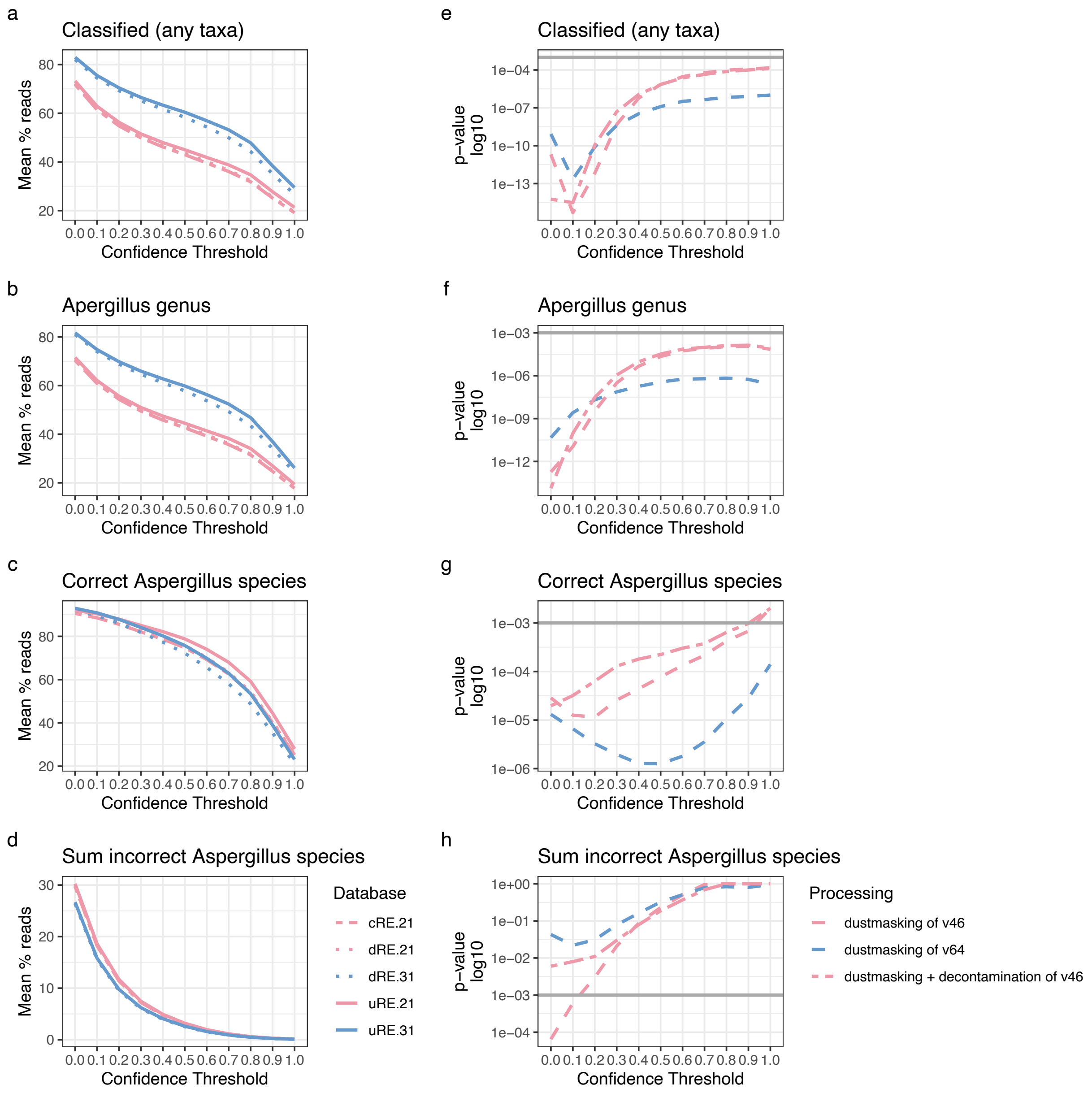

Supplementary Figure 5. Assessing the impact of genomic dustmasking and decontamination on taxonomic classification in immunocompromised control samples

The line plots in **a-d.** depict the average across our 54 separate *Aspergillus* simulated Illumina datasets remained unclassified, observed at various CTs. The metrics include **a.** overall classification, **b.** *Aspergillus* genus level, **c.** correct *Aspergillus* species, and **d.** incorrect species. **e-h.** To assess the impact of dustmasking alone and dustmasking plus decontamination (i.e. cleanup) on these four metrics, we conducted a paired one-tailed t-test with Bonferroni correction between the percentage of reads classified in a database that underwent dustmasking and/or decontamination and their unaltered counterpart (uRE.21 and uRE31). Each data point in **e-h.** shows the outcome of this t-test, with the significance threshold at p = 0.001 indicated by a gray horizontal line.

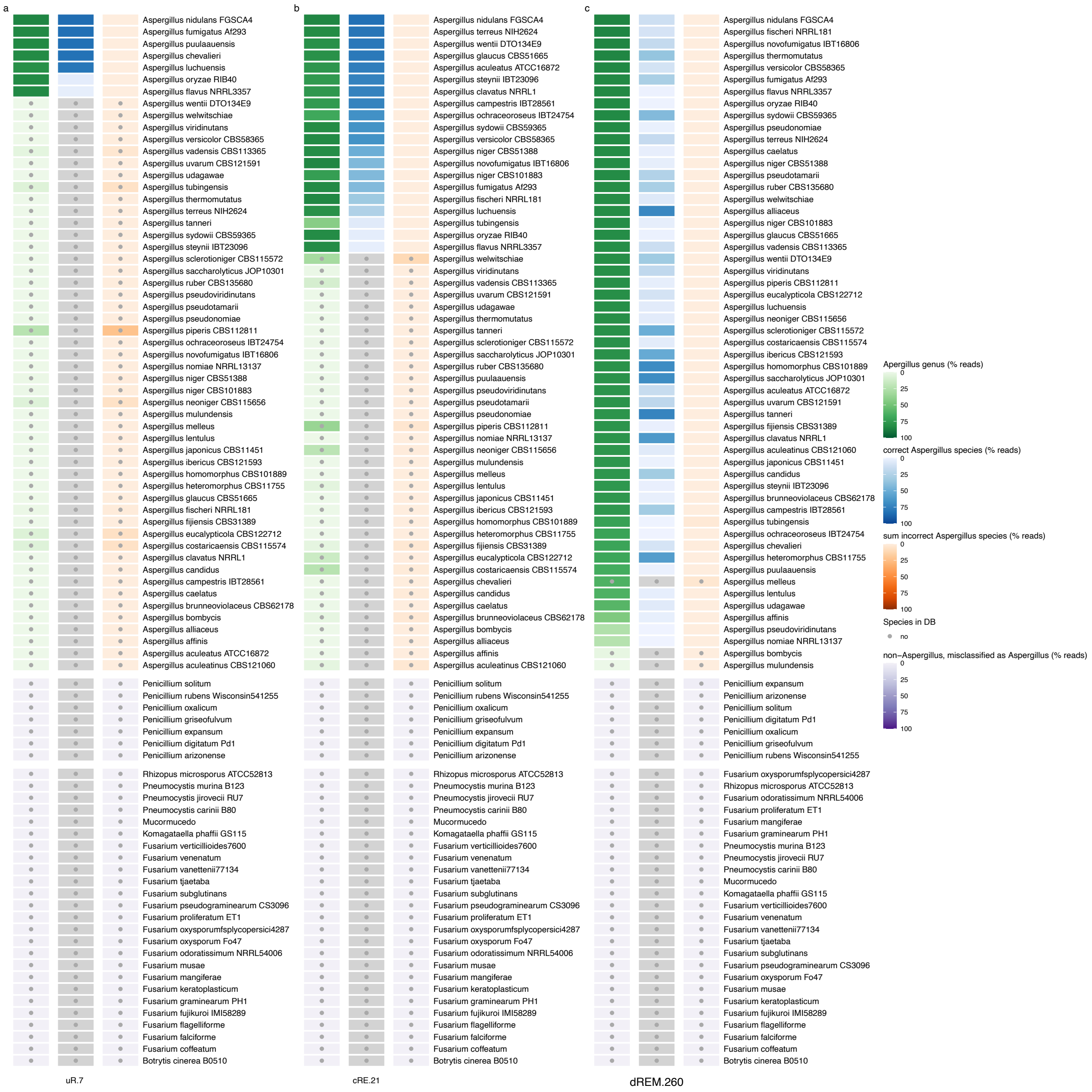

Supplementary Figure 6. Heatmap taxon detection simulated fungal datasets using uR.7, cRE.21, and dREM.260 databases

Classification results of simulated fungal datasets, including *Aspergillus* (n=54), *Penicillium* (n=7), and other clinically relevant species (n=25), using three distinct databases: **a.** uR.7, **b.** cRE.21, and **c.** dREM.260, all with a CT at 0.8. For the *Aspergillus* simulated set, percentages of reads assigned to the *Aspergillus* genus (green), correct *Aspergillus* species (blue), or incorrect *Aspergillus* assignments (purple) are presented. In non-*Aspergillus* simulations, the percentage of misclassified reads to either the *Aspergillus* genus or species is indicated in orange. The datasets include simulated species present in the classification database, or absent (marked by gray dots). The y-axis lists *Aspergillus* species sorted based on their percentage classified reads at the species level (uR.7, cRE.21) or genus level (dREM.260).

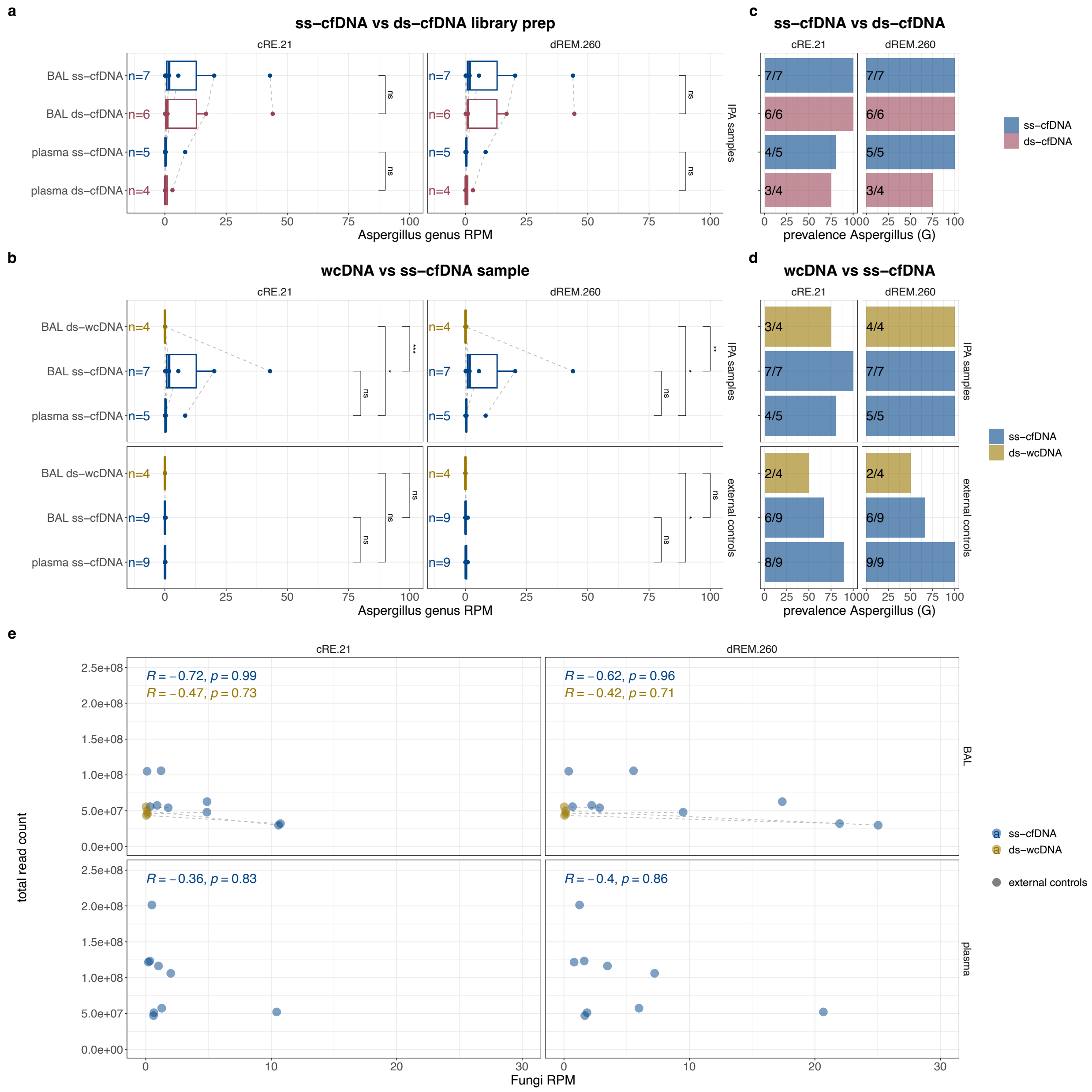

Supplementary Figure 7. Influence of library preparation on *Aspergillus* genus detection and sequence depth on fungal counts

a-b. *Aspergillus* fractional abundance in RPM, emphasizing **a.** ss- versus ds-ligation and b. ds-wcDNA versus ss-cfDNA sequencing in IPA (top row) and external control samples (bottom row) when employing the cRE.21 (left) and d.REM.260 (right) database. The boxplot bounds represent the 25th and 75th percentiles (minimum and maximum), with the center line indicating the median. Whiskers extend to 1.5 times the interquartile range. Statistical significances between library preparation and/or sample types are evaluated by one-tailed Wilcoxon rank test with Bonferroni correction (*, $p \leq 0.05$; **, $p \leq 0.01$; ***, $p \leq 0.001$; ns, $p > 0.05$). **c-d.** Barplot of *Aspergillus* genus detection prevalence using cRE.21 (left), or dREM.260 (right) databases, emphasizing **c.** ss- versus ds-ligation and **d.** ds-wcDNA versus ss-cfDNA sequencing. **e.** Scatter plot with Pearson correlation between the total read count and fractional fungal abundance (RPM) in external control samples. Pearson correlation test (*R*, one-tailed) with a $p > 0.05$ indicated there was no positive correlation between the two variables for cRE.21 (left) and dREM.260 (right) databases. BAL samples that underwent both ss-cfDNA and ds-wcDNA preparation are connected by a gray dashed line.

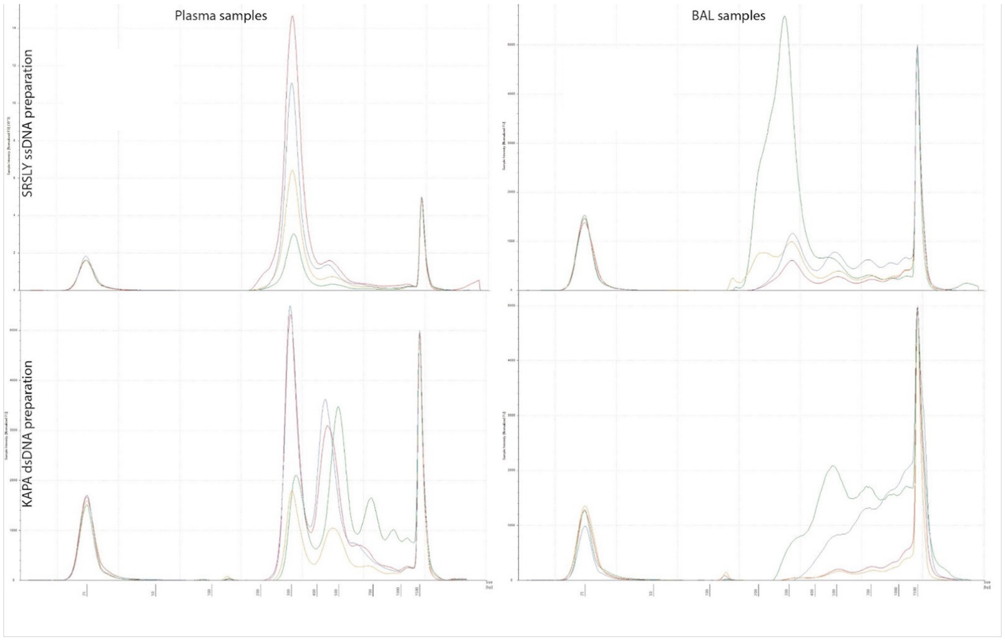

Supplementary Figure 8. Length profile analysis of ssDNA-ligated sequencing libraries in BAL and plasma samples using TapeStation

This figure illustrates the electropherogram (TapeStation 2200, D1000HS kit) capturing the length profiles of sequencing libraries from plasma and BAL fluid samples. The libraries were generated using ss-cfDNA (SRLY ssDNA preparation, top row) or ds-cfDNA (KAPA dsDNA preparation, bottom row), providing insights into the diverse profiles associated with each preparation method. For each electropherogram profile, x-axis represents the estimated fragment lengths (in bp), y-axis represents relative abundance, with reference peak at 25 bp and 1500 bp.

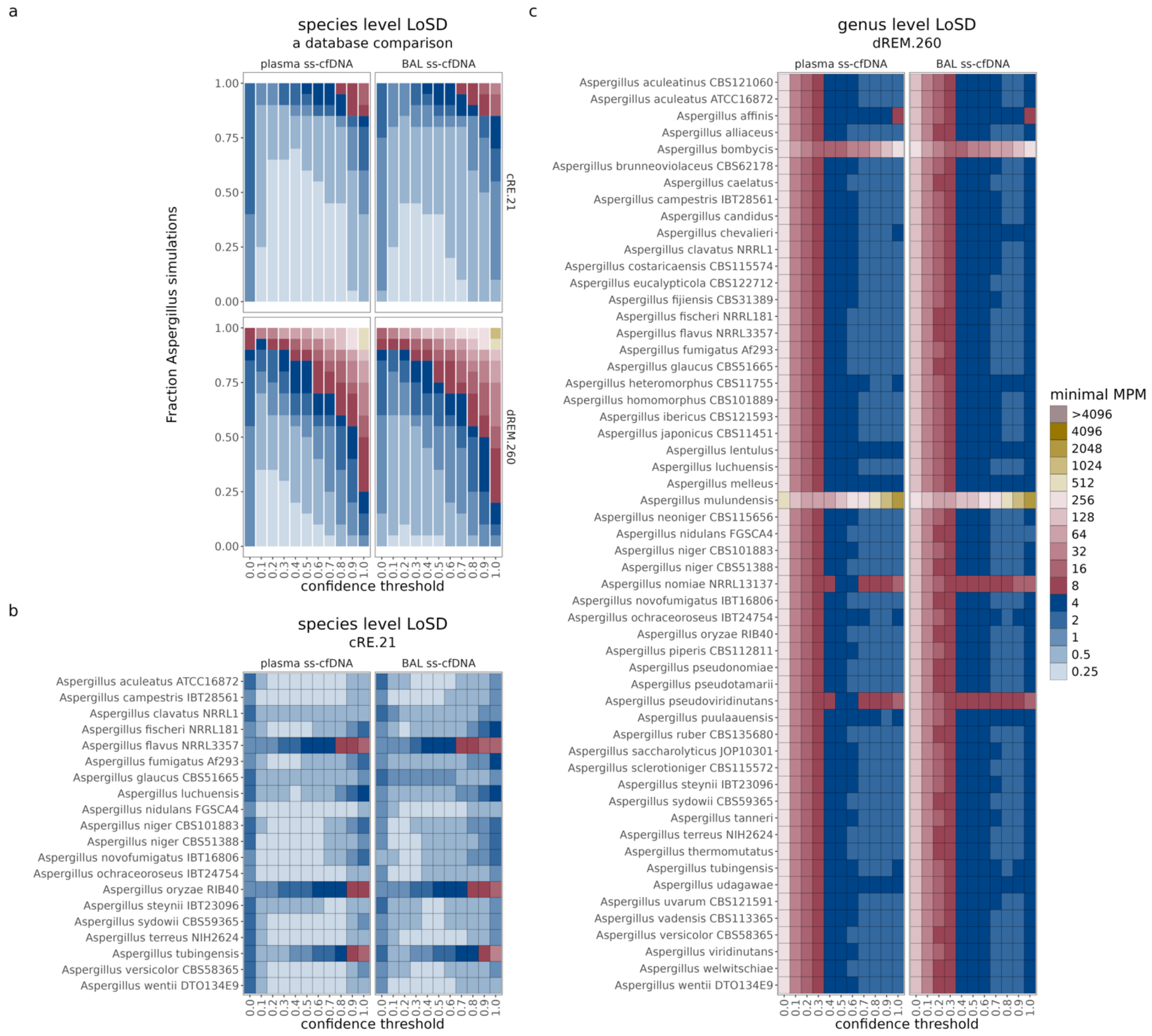

Supplementary Figure 9. LoSD per simulated *Aspergillus* dataset

Results of LoSD analysis aiming to compute the theoretical minimum fraction of *Aspergillus* molecules necessary for the detection of significantly elevated *Aspergillus* taxon above the control background noise. Fisher's exact test was employed to determine significant differences in *Aspergillus* **a-b.** species and **c.** genera count compared to our external control samples, with a mean p ≤ 0.001 considered statistically significant. **b-c.** The categorical heatmap depicts the computed limits of significance in MPM at a sequencing depth of 70 million for each *Aspergillus* simulation (y-axis; n=54) for plasma ss-cfDNA (left) or BAL ss-cfDNA (right), either **b.** at the species level (mediated by the cRE.21 database) or **c.** at the genus level (mediated by the dREM.260 database) with variable CTs from 0.0-1.0 (x-axis).

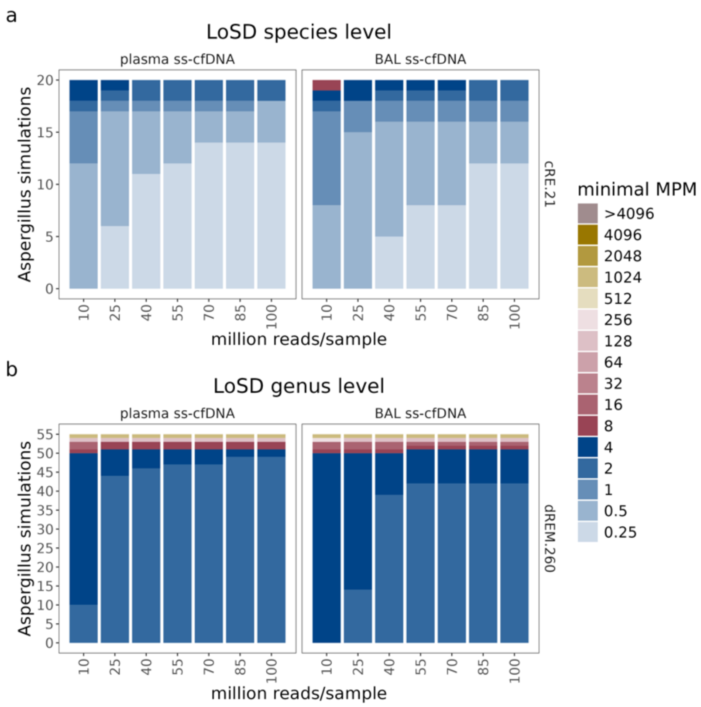

Supplementary Figure 10. Impact of sequencing depth on LoSD in simulated *Aspergillus* datasets

Variable sequencing depth results in different minimal MPM required for *Aspergillus* detection above the control background noise, i.e. LoSD. Barplots showing cumulative counts of simulated *Aspergillus* species. Fisher's exact test assessed significant differences at *Aspergillus* **a.** species level in 20 simulated *Aspergillus* species (mediated by the cRE.21 database) and **b.** genus level in 54 simulated *Aspergillus* species (mediated by the dREM.260 database), compared to external control samples, with a mean $p \leq 0.001$ considered statistically significant. The categorical heatmap illustrates computed limits of significance between 0.25 and >4096 molecules per million (MPM) at sequencing depths ranging between 10 and 100 million reads per sample (x-axis) for each Aspergillus simulation (n=54).

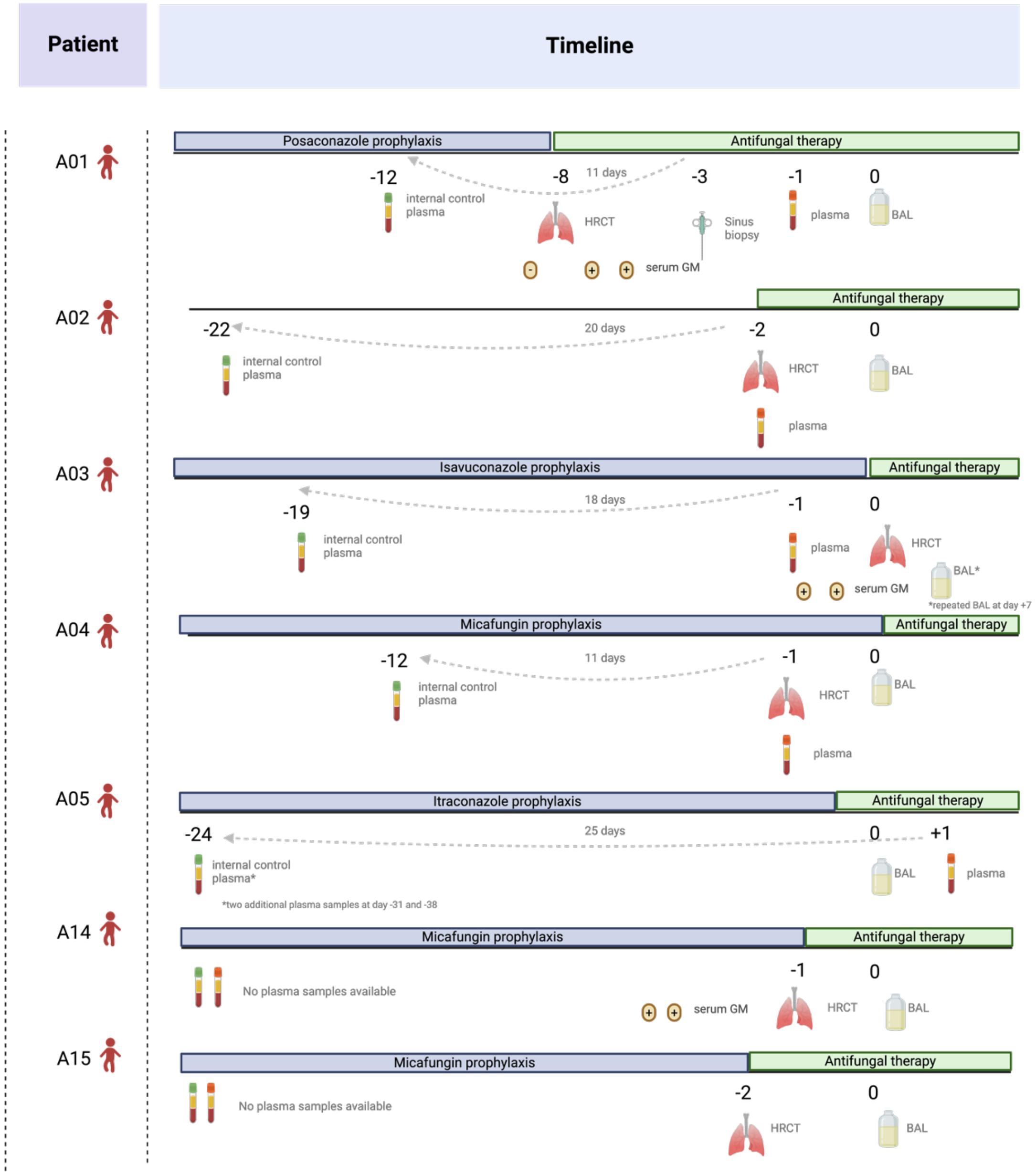

Supplementary Figure 11. Timeline clinical work-up of probable aspergillosis cases including antifungal treatment in relation to sample retrievement

Illustration of clinical timeline of all seven probable IPA patients A01-A05, A14-A15, detailing antifungal treatment, the timing of various diagnostic workups and retrieval of blood plasma. In our proof-of-principle study, both a plasma and a BAL fluid sample were included for each patient. An additional internal control plasma sample was included, acquired between 11-25 days preceding the diagnostic workup. Illustrations was created using BioRender (https://BioRender.com/i68f284).

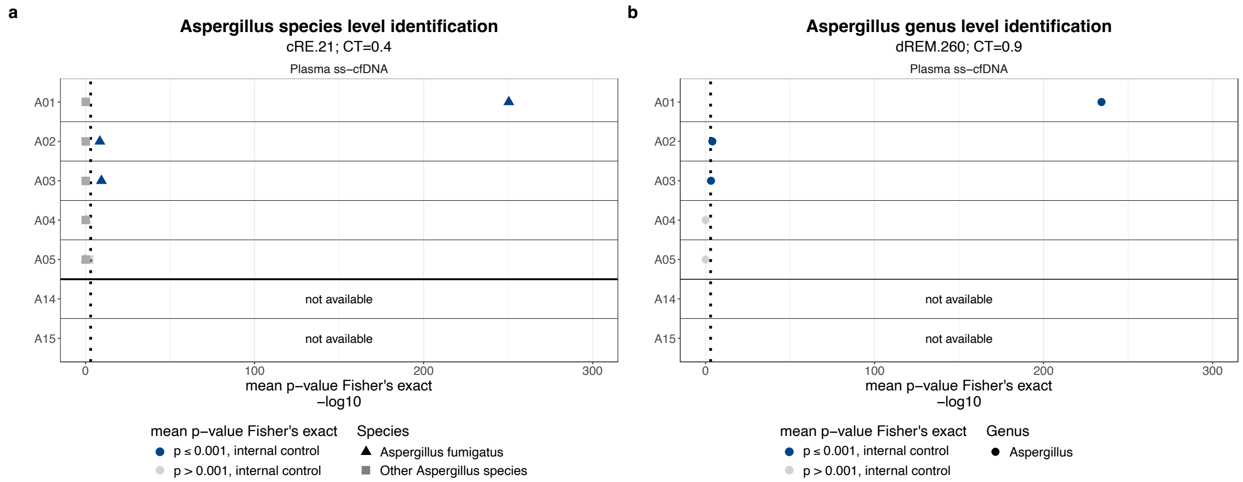

Supplementary Figure 12. Elevated *Aspergillus* levels in plasma samples with probable IPA compared to internal controls

The one-tailed Fisher's exact test was employed to assess the fractional abundance of *Aspergillus* in ss-cfDNA NGS plasma samples from patients with probable IPA. In **a-b** this comparison was made against an internal control plasma sample from the same patients, which had been collected at an earlier time point (for schematic see Supplementary Fig 11; for details see *Methods*). Comparison was made both at the **a.** species level, using the cRE.21 (CT=0.4), and **b.** at the genus level, using the dREM.260 (CT=0.9) (see *Methods* for details).

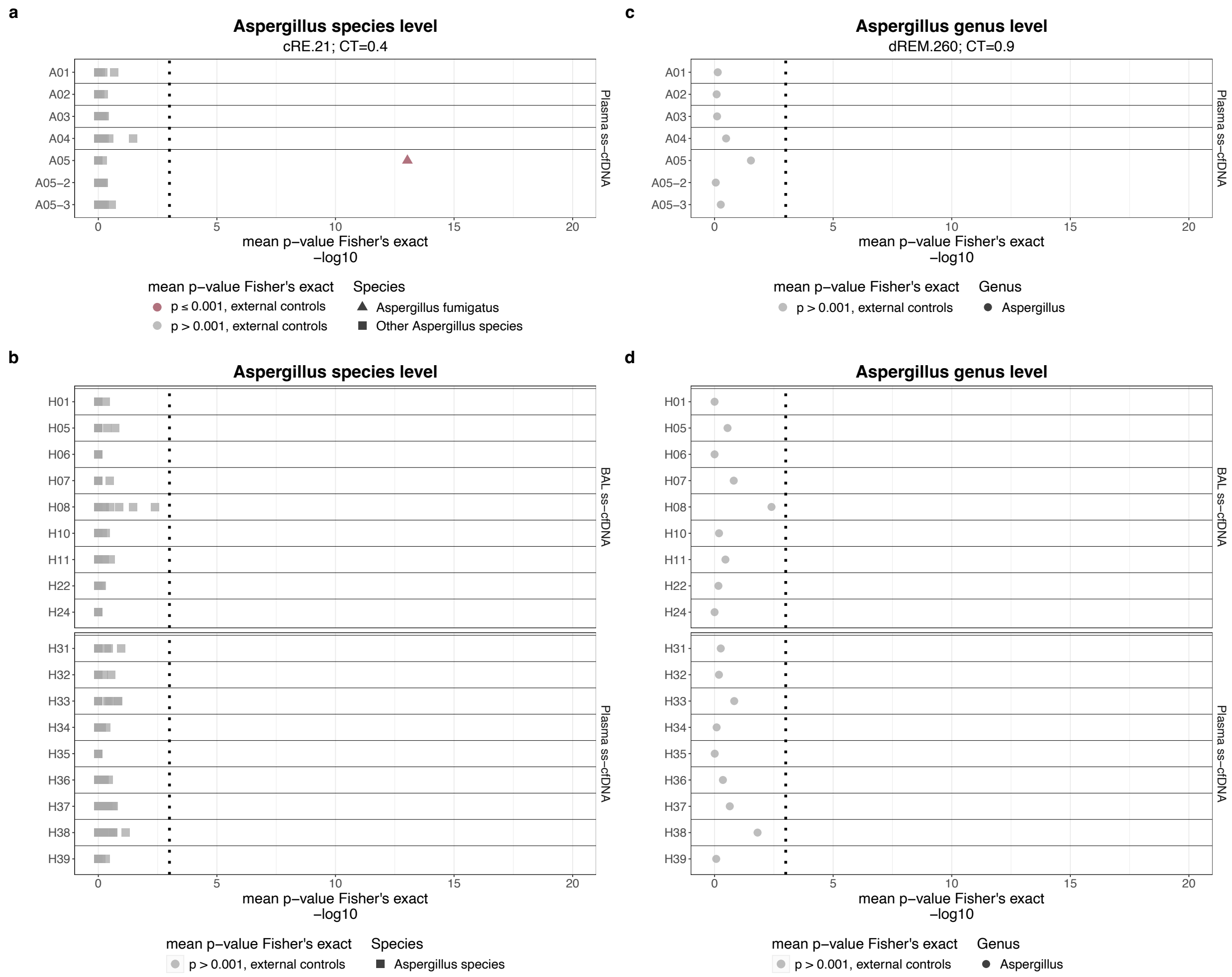

Supplementary Figure 13. ***Aspergillus*** **levels in control ss-cfDNA NGS samples**

The one-tailed Fisher's exact test was utilized to compare the fractional abundance of *Aspergillus* in a given sample to a control set of samples. Comparison entails ss-cfDNA NGS read counts of **a-b.** *Aspergillus* at the species level while employing the cRE.21 (CT=0.4) plus **c-d.** *Aspergillus* at the genus level while employing the dREM.260 (CT=0.9). We employed the one-tailed Fisher's exact test to compare **a,c.** each internal control sample (derived from a IPA patient) against external control samples (derived from immunocompromised patients), as well as to compare **b,d.** each external control sample against all other external control samples (leave-one-out principle). Dot plots display the mean -log10-transformed computed p-values, with the significance threshold set at p=0.001 indicated by a vertical dotted line. Instances exceeding the significance threshold are highlighted in red.

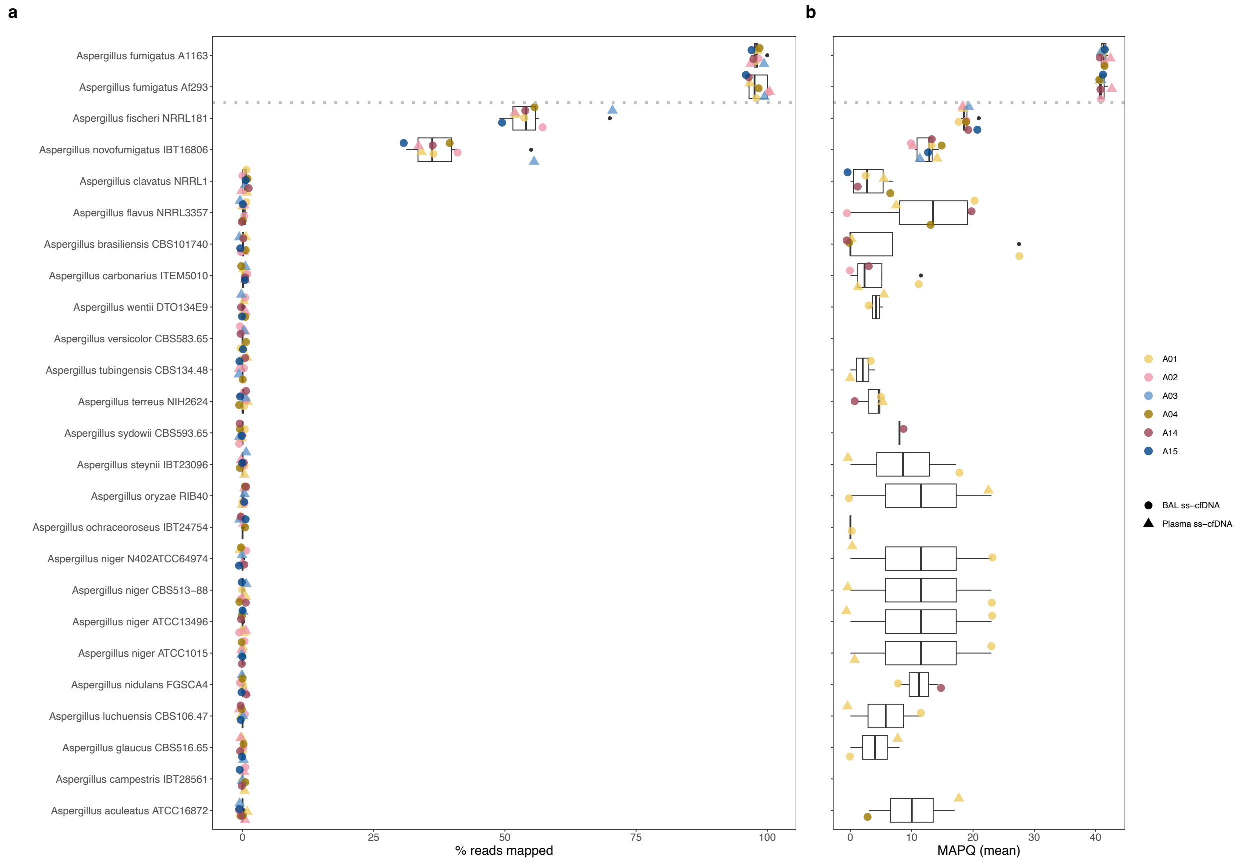

Supplementary Figure 14. Alignment *A. fumigatus* classified reads to *Aspergillus* genomes integrated in the cRE.21 database

Reads classified as *A. fumigatus* in IPA patient samples with a significantly heightened *A. fumigatus* ss-cfDNA abundance in their plasma (n=3) and/or BAL fluid (n=5) sample were realigned to all *Aspergillus* genomes integrated in the cRE.21 database. Boxplots showing **a.** the percentage of ss-cfDNA NGS reads aligned and **b.** the average mapping quality (MAPQ) of the aligned reads. Each datapoint represents a sample. The boxplot bounds in **a-b.** represent the 25th and 75th percentiles (minimum and maximum), with the center line indicating the median. Whiskers extend to 1.5 times the interquartile range.

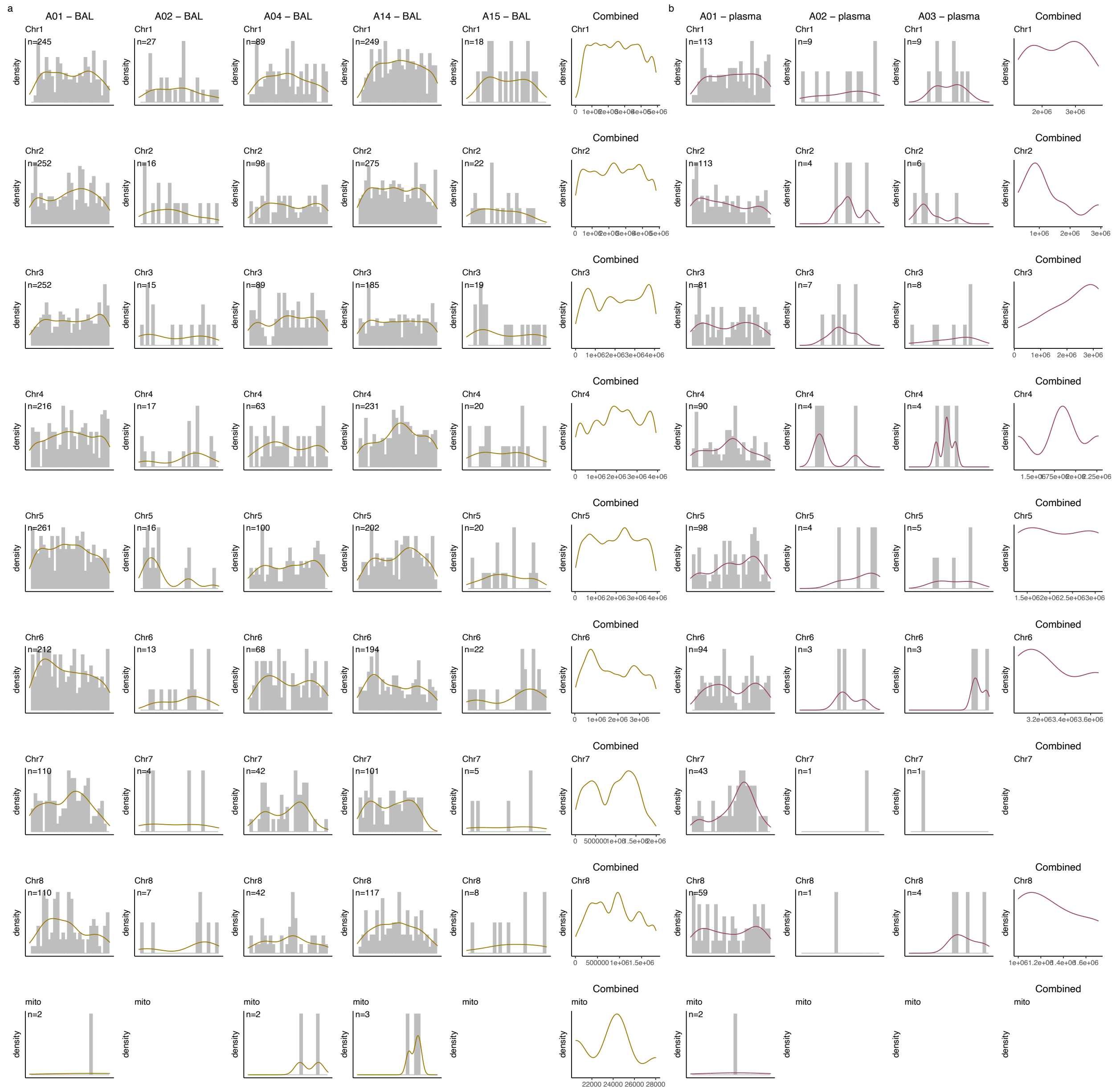

Supplementary Figure 15. Read distribution of *A. fumigatus* classified reads to the genome of *A. fumigatus* strain Af293

Histogram and density analysis of ss-cfDNA NGS reads, categorized using the cRE.21 at CT=0.4, and aligned to the *A. fumigatus* reference genome. Sequence reads obtained from a patient exhibiting notably elevated *A. fumigatus* ss-cfDNA levels in their **a.** BAL fluid and/or **b.** plasma were re-mapped to the genome of *A. fumigatus* strain Af293. The alignment was systematically displayed across varied chromosomes (rows), with each column symbolizing a unique IPA patient sample. The count of aligned reads is provided for each subplot in the upper-left corner (n). The alignment of all **a.** BAL and **b.** plasma samples are summarized in yellow and dark-red respectively, and demonstrates uniformity across different chromosomes.

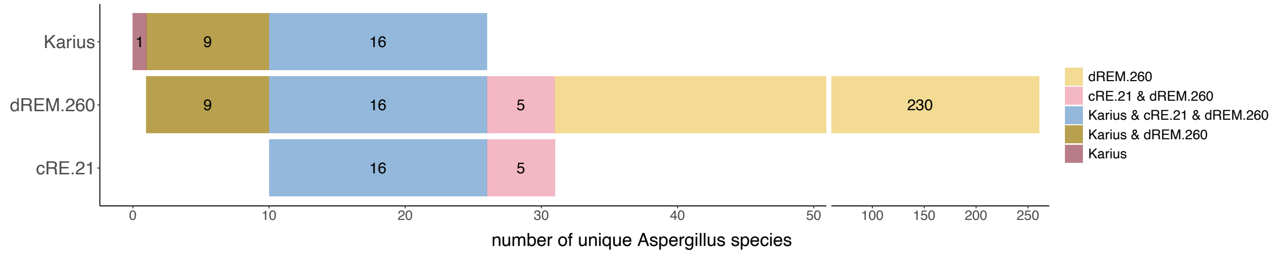

Supplementary Figure 16. Comparative analysis of pathogen list concordance with cRE.21 and dREM.260 databases

The bar plot visually depicts the (dis)concordance in *Aspergillus* species quantity between the pathogen list from Karius and the ones in the cRE.21 and dREM.260 databases.
